# *VIP1* and Its Homologs Are Not Required for *Agrobacterium*-Mediated Transformation, but Play a Role in *Botrytis* and Salt Stress Responses

**DOI:** 10.3389/fpls.2018.00749

**Published:** 2018-06-12

**Authors:** Rachelle Lapham, Lan-Ying Lee, Daisuke Tsugama, Sanghun Lee, Tesfaye Mengiste, Stanton B. Gelvin

**Affiliations:** ^1^Department of Biological Sciences, Purdue University, West Lafayette, IN, United States; ^2^Laboratory of Crop Physiology, Research Faculty of Agriculture, Hokkaido University, Sapporo, Japan; ^3^Department of Botany and Plant Pathology, Purdue University, West Lafayette, IN, United States

**Keywords:** *Agrobacterium*-mediated transformation, fungal tolerance, protein localization, protein–protein interactions, salt tolerance, VIP1 transcriptome, VirE2

## Abstract

The bZIP transcription factor VIP1 interacts with the *Agrobacterium* virulence protein VirE2, but the role of VIP1 in *Agrobacterium*-mediated transformation remains controversial. Previously tested *vip1-1* mutant plants produce a truncated protein containing the crucial bZIP DNA-binding domain. We generated the CRISPR/Cas mutant *vip1-2* that lacks this domain. The transformation susceptibility of *vip1-2* and wild-type plants is similar. Because of potential functional redundancy among VIP1 homologs, we tested transgenic lines expressing VIP1 fused to a SRDX repression domain. All VIP1-SRDX transgenic lines showed wild-type levels of transformation, indicating that neither VIP1 nor its homologs are required for *Agrobacterium*-mediated transformation. Because VIP1 is involved in innate immune response signaling, we tested the susceptibility of *vip1* mutant and *VIP1-SRDX* plants to *Pseudomonas syringae* and *Botrytis cinerea*. *vip1* mutant and *VIP1-SRDX* plants show increased susceptibility to *B. cinerea* but not to *P. syringae* infection, suggesting a role for VIP1 in *B. cinerea*, but not in *P. syringae*, defense signaling. *B. cinerea* susceptibility is dependent on abscisic acid (ABA) which is also important for abiotic stress responses. The germination of *vip1* mutant and *VIP1-SRDX* seeds is sensitive to exogenous ABA, suggesting a role for VIP1 in response to ABA. *vip1* mutant and *VIP1-SRDX* plants show increased tolerance to growth in salt, indicating a role for VIP1 in response to salt stress.

## Introduction

Virulent strains of the soil bacterium *Agrobacterium tumefaciens* cause the tumorigenic disease crown gall. *Agrobacterium*-mediated plant genetic transformation involves mobilization of transferred-DNA (T-DNA) and five virulence proteins (VirD2, VirE2, VirE3, VirD5, and VirF) from the bacterium into a plant cell (Gelvin, 2003, 2012).

The effector protein VirE2 has non-specific single-stranded DNA-binding activity and is thought to coat single-stranded T-DNA (T-strands) after entry into the plant cell (Citovsky et al., 1992), protecting T-strands from nucleolytic degradation (Yusibov et al., 1994; Rossi et al., 1996). In addition to this structural role, VirE2 interacts with a number of plant proteins including VirE2-interacting protein 1 (VIP1; Tzfira et al., 2001) and VIP2 (Anand et al., 2007). VIP1, a bZIP transcription factor which is a target of the mitogen-activated protein kinase 3 (MPK3), is thought to be involved in plant defense responses (Djamei et al., 2007; Pitzschke et al., 2009). Phosphorylation of VIP1 on serine 79 by MPK3 results in the import of VIP1 into the plant nucleus (Djamei et al., 2007). VIP1 may subsequently bind to VIP1 response elements (VREs) to activate transcription of its target genes (Pitzschke et al., 2009). VIP1 may also be involved in sulfur utilization, starch accumulation, osmosensory signaling, and touch-induced root waving (Ishida et al., 2004; Wu et al., 2010; Tsugama et al., 2012, 2014, 2016; Chen et al., 2015).

The importance and role of VIP1 in *Agrobacterium*-mediated transformation are controversial (Tzfira et al., 2001; Shi et al., 2014). Previous studies using transgenic tobacco lines expressing antisense constructs targeting *VIP1*, and the *Arabidopsis thaliana* T-DNA insertion mutant *vip1-1*, found that these plants showed decreased stable transformation compared to that of wild-type plants (Tzfira et al., 2001; Li et al., 2005). Overexpression of *VIP1* in tobacco resulted in increased transformation susceptibility, suggesting that VIP1 plays a role in transformation (Tzfira et al., 2001). However, quantitative transformation assays with the *vip1-1* mutant and with 59 *A. thaliana VIP1* overexpressing lines showed no effect on transformation susceptibility (Shi et al., 2014), suggesting that VIP1 is not important for *Agrobacterium*-mediated transformation.

Previous studies indicated that β-glucoronidase-(GUS) or YFP-tagged VirE2 localizes to the plant nucleus (Citovsky et al., 1992, 1994; Tzfira and Citovsky, 2001). However, other studies showed exclusively cytoplasmic localization of VirE2 (Bhattacharjee et al., 2008; Grange et al., 2008; Lee et al., 2008; Sakalis et al., 2013; Shi et al., 2014). VirE2 possesses a weak putative nuclear localization signal (NLS) sequence which does not bind strongly to importin α protein (Citovsky et al., 1994; Chang et al., 2014). Early work indicated that VirE2 does not interact with *Arabidopsis* importin alpha-1 (IMPa-1, also known as AtKAPα) in yeast (Ballas and Citovsky, 1997), although Bhattacharjee et al. (2008) subsequently detected such interactions in yeast, *in planta*, and *in vitro.* However, VirE2-IMPa-1 complexes remained cytoplasmic in plants (Lee et al., 2008). VirE2 nuclear import has been attributed to its interaction with VIP1 (Tzfira et al., 2001), a protein that localizes to both the cytoplasm and the nucleus (Djamei et al., 2007; Shi et al., 2014). Activation of VIP1 by MPK3 and subsequent binding of phosphorylated VIP1 to VirE2 may facilitate nuclear localization of VIP1-VirE2-T-strand complexes (the Trojan-horse model; Djamei et al., 2007).

The *vip1-1* mutant still produces ∼80% of the VIP1 protein, including the crucial bZIP DNA-binding domain (Li et al., 2005). Because this domain may be important for function, we used CRISPR technology to generate a homozygous mutant, *vip1-2*, that produces a smaller protein lacking the bZIP domain. Transient and stable transformation assays indicated no effect of this mutation on *Agrobacterium*-mediated transformation. Furthermore, transformation assays of single and multiple null mutant lines of *VIP1* homologs, and transgenic lines overexpressing VIP1 fused to a modified EAR-like motif repression domain (SRDX; Hiratsu et al., 2002), also failed to show any major effect on transformation. We therefore conclude that *VIP1* and its homologs are not required for *Agrobacterium-*mediated transformation. However, *VIP1* may be important for defense responses against the fungus *Botrytis cinerea*, for abscisic acid (ABA) signaling, and for growth under salt stress conditions.

## Materials and Methods

### Plasmids and Strain Constructions

Supplementary Tables 1, 2 list the plasmids, strains, and single-guide RNA sequences used in this study. To make *VIP1* CRISPR-Cas9 constructs, we designed three sets of sgRNA constructs targeting the *VIP1* gene within the first exon. For each set, two 20-nucleotide oligomers of target DNA sequences were synthesized with an additional GATC on the 5′ end of the sense-strand and AAAC on the 5′ end of the antisense-strand. After annealing, we cloned this double stranded oligomer into the *Bbs*I site of psgR-Cas9-At. A *Hin*dIII-*Kpn*I fragment from this plasmid (containing both sgRNA and Cas9 expression cassettes) was cloned into pCAMBIA1300 to make the plasmids pE4351, pE4352, and pE4353. These T-DNA binary vectors were introduced by electroporation into *A. tumefaciens* GV3101 (Van Larebeke et al., 1974) to generate *A. tumefaciens* At2115, At2116, and At2117, respectively.

To make the *vip1-2*-Venus (out of frame) fusion construct, we cloned the *vip1-2* RT-PCR product into the *Sma*I site of pBluescript KS+ to create pE4443. *Bgl*II and *Bam*HI sites were used to remove the *vip1-2* cDNA fragment, which was ligated to the *Venus* gene in pE3857 to produce pE4451 (confirmed by sequencing; Supplementary Table 1).

To create the *vip1-2*-GUS-Venus fusion construct, the nucleotides encoding the first 145 amino acids of *vip1-2* was amplified by PCR using the primers VIP1-*Bgl*II-FP1 and *vip1-2* peptide, flanked by *Bgl*II and *Bam*HI sites, respectively. The PCR product was cloned into the *Sma*I site of pE886 to create pE4516. The *Bgl*II-*Bam*HI fragment was made blunt with Klenow fragment of DNA polymerase (New England Biolabs) and cloned into the *Bgl*II site of pE3835 to make pE4521. A plasmid (pE4517) containing the VIP1-GUS-Venus fusion was made by cloning the *Bam*HI-*Bgl*II fragment from pE3857 into the *Bgl*II site of pE3835.

To create the inducible *VIP1* overexpression construct, we excised an *Sph*I-*Xho*I fragment, containing the LexA operator and a minimal Cauliflower Mosaic Virus 35S promoter, from pER8 (Zuo et al., 2000). The fragment, made blunt using Klenow fragment of DNA polymerase, was ligated to pE3542 digested with *Age*I and *Xho*I and made blunt. The resulting plasmid, pE3542, is a pSAT1-derived cloning vector used to generate β-estradiol-inducible gene constructions.

To generate the T-DNA binary vector into which the inducible gene constructions were placed, we ligated a blunted *Sbf*I-*Nco*I fragment containing the XVE expression cassette and a Pnos-partial *hptII* gene into the blunted *Swa*I-*Nco*I site of pE4145 (pPZP-RCS-*hptII*) to make pE4216. We then ligated a *Nco*I fragment containing part of the *hptII* gene from pER8 to the *Nco*I site of pE4216 to generate a complete *hptII* gene (pE4215). pE4215 is a T-DNA binary vector containing the XVE and *hptII* expression cassettes and a *AscI* site into which the inducible gene expression cassette can be cloned.

The *Swa*I-*Not*I fragment containing the *VIP1* gene was removed from the plasmid pE4132 and cloned into the *Sma*I and *Not*I sites of the β-estradiol inducible promoter plasmid pE3542, making pE4275. pE4275 was then digested with *Asc*I and the inducible *VIP1* fragment was cloned into the *Asc*I site of the binary vector pE4215 to create pE4288. pE4288 was introduced by electroporation into *A. tumefaciens* GV3101 (Van Larebeke et al., 1974) to generate *A. tumefaciens* At2082.

### Generation and Screening of *VIP1* CRISPR/Cas9 and Inducible *VIP1* Transgenic *A. thaliana* Plants

Wild-type Col-0 ecotype *A. thaliana* plants were transformed by *A. tumefaciens* At2115, At2116, At2117, or At2082 using a flower dip protocol (Clough and Bent, 1998). T0 generation seeds harvested from transformed plants were surface sterilized for 15 min using a 50% Bleach and 0.1% sodium dodecylsulfate (SDS) before washing 5 times with sterile water. After incubation overnight at 4°C, the seeds were plated on solidified Gamborg’s B5 medium (Caisson Labs) containing 100 μg mL^-1^ Timentin and 20 μg mL^-1^ hygromycin. The seeds were incubated at 23°C using a 16/8-h light/dark cycle. Hygromycin-resistant seedlings (T1 generation) were transplanted to soil and grown under the same temperature and light conditions. Seeds were harvested from each T1 plant and T2 generation plants grown in soil. For the *VIP1* CRISPR/Cas9 plants, DNA isolated from leaves of individual T2 plants was used to PCR-amplify a region surrounding the sgRNA target site using primers listed in Supplementary Table 2. The PCR products were analyzed for mutations using a T7 endonuclease I (New England Biolabs) mismatch assay (Babon et al., 2003). Mutations were confirmed by sequencing. For inducible *VIP1* plants, seeds were harvested from the T2 generation plants and selected on hygromycin. Seeds from homozygous plants (100% progeny surviving on selection) were used for future experiments.

### *VIP1* Induction in the Presence and Absence of *Agrobacterium*

The T3 generation inducible *VIP1* seedlings were germinated on B5 medium containing 100 μg mL^-1^ Timentin and 20 μg mL^-1^ hygromycin. After 2 weeks, the seedlings were transferred to plates containing B5 medium only which were placed vertically in racks to allow for root tissue to grow on the surface of the medium. After 7 days, B5 liquid medium containing 1 μM of β-estradiol suspended in DMSO (induction solution) or B5 with DMSO only (control solution) was pipetted onto the plates until a thin layer of liquid covered the root tissue. To determine differential gene expression in the presence of *Agrobacterium*, cells of *A. tumefaciens* A136 (lacking a Ti-plasmid) were suspended in either induction or control solution at a concentration of 10^8^ cells mL^-1^. The roots were incubated in the treatment solution for either 3 or 12 h before cutting the roots from the stems using a razor blade, rinsing with sterile water, dabbing them dry with a paper towel, and freezing them in liquid nitrogen. For each treatment, the root tissue was pooled from 30 individual plants. The tissue was stored at -80°C.

### Preparation of Samples for Quantitative RT-PCR

The RNA was isolated from the root tissue of untreated, non-induced, induced, non-induced in the presence of *Agrobacterium*, and induced in the presence of *Agrobacterium* after 0, 3, and 12 h of incubation. This was done for two biological replicates of inducible *VIP1 A. thaliana* transgenic line #12 and inducible *VIP1 A. thaliana* transgenic line #8.

A total of 1.45 μg of total RNA was treated with Ambion DNase I (Thermo Fisher Scientific) and SuperScriptIII reverse transcriptase (Thermo Fisher Scientific) was used to synthesize cDNA according to the manufacturer’s protocols. Quantitative RT-PCR was performed with a Roche LightCycler 96 using FastStart Essential Green Master reagents (Roche). Primers used to amplify the genes are described in Supplementary Table 2. Data were analyzed using the LightCycler 96 software, REST^1^ 2009 software, and Microsoft Excel.

### Phenotypic Characterization of *vip1-2* Plants

Homozygous *vip1-2* and wild-type Col-0 plants were grown on soil at 23°C in a chamber with a 16/8-h light/dark cycle. After germination, plants were thinned to one plant per pot and photos were taken every 2–3 days throughout growth. Rosette, leaf, and flower bolt sizes were measured using image processing software and statistical analysis was performed using a Student’s *t*-test.

### Isolation and Transfection of *Arabidopsis* and Tobacco BY-2 Protoplasts

Protoplasts were isolated from leaves of wild-type (ecotype Col-0) and *vip1-2 A. thaliana* plants and tobacco BY-2 cells and transfected as described in Lee et al. (2012). pE3170 (mRFP-nuclear marker) was co-transfected into protoplasts with the appropriate clones. Protoplasts were imaged 16 h after transfection using a Nikon A1R Confocal Laser Microscope System as described in Shi et al. (2014).

### *Agrobacterium*-Mediated Transient and Stable Transformation Assays

*Agrobacterium thaliana* lines tested in this study are listed in Supplementary Table 3. Homozygous lines for the annotated T-DNA insertions were confirmed by PCR (primer sequences listed in Supplementary Table 2). Roots from 20-day-old *A. thaliana* plants grown in baby food jars containing sterile Gamborg’s B5 medium were cut into 3–5 mm segments. Root segments were assayed as described in Tenea et al. (2009). *A. tumefaciens* At849 [GV3101 containing pBISN1 (Narasimhulu et al., 1996)] was used for transient transformation assays, whereas *A. tumefaciens* A208 was used for stable transformation. Three replicates were performed for each experiment and root segments were pooled from 6 to 10 plants for each replicate. A total of 80 or more root segments were scored for each data point. Statistical analysis was performed using a Student’s *t*-test.

### Quantitative RT-PCR of *vip1-2*

The RNA was isolated from leaf tissue harvested from 3-week-old plants grown on soil using TriZol^2^ reagent. For each sample, 1 μg of total RNA was treated with Ambion DNase I (Thermo Fisher Scientific) and SuperScriptIII reverse transcriptase (Thermo Fisher Scientific) was used to synthesize cDNA according to the manufacturer’s protocols. Real-time PCR was performed with a Roche LightCycler 96 using the FastStart Essential Green Master reagents (Roche). Primers used to amplify the 3′ end of the *VIP1* transcript are described in Supplementary Table 2. Data were analyzed using the LightCycler 96 software, REST^3^ 2009 software, and Microsoft Excel.

### *Botrytis cinerea* and *Pseudomonas syringae* Pathogenesis Assays

Five-week-old *A. thaliana* wild-type (Col-0), *vip1-1, vip1-2*, and *VIP1-SRDX* Line #11 leaves (from plants grown on soil) were inoculated with 5 μL of *B. cinerea* at a concentration of 1.0 × 10^5^ spores/mL. Lesion size was measured 3 days after infection and averaged over 18 leaves per genotype (36 leaves for Col-0). Standard error was calculated over two separate experiments and a Student’s *t*-test was used to test for significant differences.

Five-week-old *A. thaliana* wild-type (Col-0), *vip1-1, vip1-2*, and *VIP1-SRDX* Line #11 leaves (from plants grown on soil) were syringe-inoculated with *P. syringae pv. tomato* DC3000 (virulent) or *P. syringae pv. tomato* DC3000 *hrcC* (avirulent) at an optical density (A_600_) of 0.001 and 0.005, respectively. Bacterial growth was determined at 0 and 4 days after infection by isolating bacteria from six leaf disks for each plant and plating a dilution series to calculate the number of colony-forming units (cfu) per square centimeter of leaf material. Standard error was calculated over three replicates and a Student’s *t*-test was used to test for significant differences.

### ABA and Hyper-Osmotic Germination and Root Growth Assays

Seeds were plated onto ½ MS 1% sucrose medium containing 0, 0.3, or 0.5 μM ABA or onto MS 2% sucrose medium containing 0, 50, 75, or 100 mM NaCl. A total of 25 seeds per genotype were placed on each plate with two (ABA) or four (NaCl) plates prepared for each treatment. The plates were incubated in a 23°C chamber with a 16/8-h light/dark cycle. Germinated seeds were scored 8 days after plating. Any seed with a radicle protruding was considered to have germinated. The number of germinated seeds was divided by the total number to calculate the percent germination and this was averaged over all the plates for each treatment. Student’s *t*-test was used to test for statistically significant differences.

To test the effect of exogenous ABA or hyper-osmotic conditions on root growth, seeds were germinated on ½ MS, 1% sucrose medium (ABA) or MS 2% sucrose medium (hyper-osmotic). The 5-day-old seedlings with a root length of ∼1 cm were transferred to ½ MS 1% sucrose containing 0, 2, or 20 μM of ABA or MS 2% sucrose with 0, 50, 75, or 100 mM of NaCl. These plates were placed vertically in racks in a 16/8-h light/dark cycle growth chamber at 23°C. A total of 11 plates were prepared for each treatment. Pictures were taken of each of the ABA and NaCl plates, 8 and 11 days (respectively), after the seedlings were transferred. Root length was determined using ImageJ software. Root length was averaged over 11 seedlings for each genotype for each treatment. The rate of growth was determined by subtracting the initial root length from the final root length divided by the number of days of growth. Student’s *t*-test was used to test for statistically significant differences.

## Results

### Generation of the *vip1-2* Mutant

**FIGURE 1:**
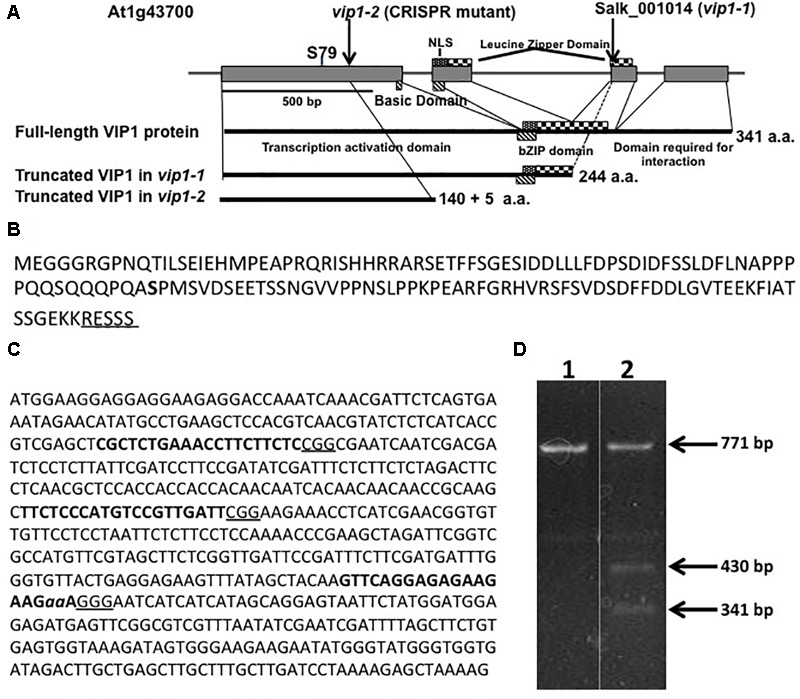
The *VIP1, vip1-1*, and *vip1-2* genes and coding regions. **(A)** Map of the *VIP1* coding region. Important protein domains are shown for the full-length and truncated VIP1-1 and VIP1-2 proteins. The C-terminal domain absent in both mutant proteins is required for VIP1 dimerization and VIP1-Histone H2A interactions (Li et al., 2005). Serine^79^, a phosphorylation site important for nuclear targeting of VIP1 (Djamei et al., 2007), is indicated. **(B)** Amino acid sequence of the VIP1-2 protein. The five amino acids shown underlined are not from the VIP1 protein, but result from the 2 bp deletion before a stop codon is reached. Serine^79^ is indicated in bold. **(C)** DNA sequence of the first exon of *VIP1*. Target sites for single-guide RNAs are highlighted in bold. The PAM sequences are underlined. The two nucleotides *aa* are deleted in the *vip1-2* mutant. **(D)** T7 endonuclease I digestion of *VIP1* PCR products (771 bp of gDNA surrounding the mutation in *vip1-2*) using wild-type gDNA as template (Lane 1) or a mixture of PCR products from both wild-type *and vip1-2* mutant gDNAs (Lane 2). The mutation in *vip1-2* creates a 2-bp mismatch generating two bands of 430 and 341 bp after T7 endonuclease cleavage.

Several laboratories have utilized the T-DNA insertion mutant *vip1-1* (SALK_001014.38.85.x) to study the role of VIP1 in *Agrobacterium*-mediated transformation and other cellular processes (Li et al., 2005; Pitzschke et al., 2009; Wu et al., 2010; Tsugama et al., 2012, 2016; Shi et al., 2014; Chen et al., 2015). However, *vip1-1* is not a transcriptional null mutant and still produces the first 244 amino acids of the 341 amino acid VIP1 protein (Li et al., 2005; Shi et al., 2014). The VIP1-1 protein lacks the C-terminal domain necessary for self-dimerization and interaction with histone H2A (Li et al., 2005; Lacroix et al., 2008), but still contains the transcriptional activation domain as well as the majority of the bZIP DNA-binding domain (**Figure 1A**). We therefore used CRISPR-Cas9 technology (Feng et al., 2013) to generate a *vip1* mutant that produces a smaller VIP1 protein (**Figure 1B**).

We designed three guide RNAs to target multiple positions in the first exon of VIP1 (**Figure 1C**). T7 endonuclease analysis (Babon et al., 2003) of numerous T2 generation transgenic *Arabidopsis* lines expressing individual guide RNAs and Cas9 failed to identify mutations using constructs targeting the two most 5′-proximal regions of the *VIP1* gene. However, the third guide RNA generated several different mutations (**Figure 1D**). DNA sequence analysis confirmed that one of these mutations resulted in a two base pair deletion, generating a premature stop codon. This mutant, *vip1-2*, encodes the first 140 amino acids of VIP1 plus five additional amino acids resulting from the frame-shift mutation. VIP1-2 lacks the bZIP DNA-binding domain, the nuclear localization signal (NLS) sequence, and the C-terminal domain important for VIP1 dimerization or interaction with histone H2A (**Figure 1A**).

### Properties of the *vip1-2* Gene and VIP1-2 Protein

RNA was isolated from homozygous *vip1-2* leaves and RT-PCR was performed to determine whether *VIP1* transcripts were still produced (Supplementary Figure 1A). Despite the presence of an early stop codon within the first exon of the *vip1-2* gene, primers set at the 3′ end of the gene amplified a product, indicating that the *VIP1* transcript was still produced. However, quantitative RT-PCR detected the *VIP1* transcript at 35% of the level found in wild-type plants (Supplementary Figure 1B). The reduced level of the *VIP1* transcripts may result from nonsense-mediated decay (Brogna and Wen, 2009). To verify that the *vip1-2* mutant cannot make full-length VIP1 protein, we fused the *vip1-2* cDNA to a Venus fluorescent protein coding sequence just before the position of the stop codon of the wild-type *VIP1* cDNA. When introduced into BY-2 cells, this cDNA should not result in fluorescence because of the premature stop codon in the *vip1-2* cDNA. Supplementary Figure 1C shows that a wild-type *VIP1*-*Venus* cDNA fusion construction could promote fluorescence in BY-2 cells. However, the *vip1-2*-*Venus* cDNA fusion construction could not.

**FIGURE 2:**
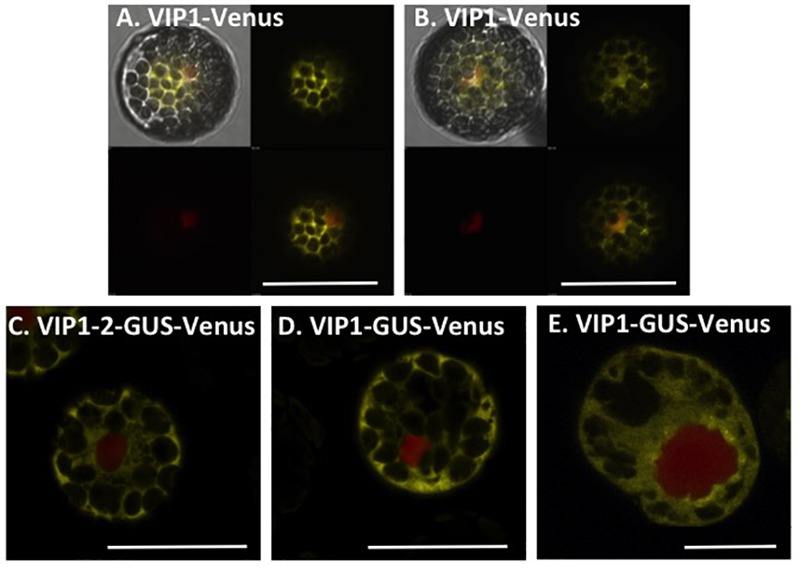
Subcellular localization of VIP1 and VIP1-2 proteins in protoplasts. Protoplasts were co-transfected with the indicated Venus-tagged constructs and a mRFP-NLS construct that marks the nucleus. A total of 16 h after transfection, the cells were imaged by confocal microscopy. VIP1-Venus localizes in both the cytoplasm and the nucleus of Col-0 **(A)** and *vip1-2* **(B)** protoplasts; VIP1-2-GUS-Venus localizes exclusively in the cytoplasm of Col-0 protoplasts **(C)**; localization of VIP1-GUS-Venus is limited to the cytoplasm of Col-0 **(D)** and tobacco BY-2 **(E)** protoplasts. In **(A,B)**, four images of the same cell are presented (clockwise from top left: merged YFP, mRFP, and DIC; YFP; YFP + mRFP; mRFP). In **(C–E)**, only the merged YFP and mRFP images are presented. Bars indicate 20 μm.

Because wild-type VIP1 protein dimerizes (Li et al., 2005), we were concerned that Venus-tagged VIP1 may interact with untagged VIP1 present in cells, and that untagged full-length VIP1 may direct the subcellular localization of the dimer complex. We therefore conducted VIP1 subcellular localization experiments in both wild-type and *vip1-2* mutant protoplasts. Wild-type VIP1-Venus fusion protein localized to both the plant cytoplasm and nucleoplasm, but not the nucleolus, of wild-type and *vip1-2* mutant *Arabidopsis* protoplasts (**Figures 2A,B**; Djamei et al., 2007; Shi et al., 2014). The VIP1-2 protein is small (16,016 Da), and even when fused to Venus would produce a protein below the nuclear exclusion limit (< 60 kDa; Dingwall and Laskey, 1991), permitting nuclear entry of a VIP1-2-Venus fusion protein by diffusion. We therefore fused the VIP1-2 protein in-frame with a GUS-Venus protein, creating a protein (111.77 kDa) that exceeds the nuclear size exclusion limit. Transfection of a plasmid containing a VIP1-2-GUS-Venus expression cassette, together with a plasmid encoding a red fluorescence protein (RFP) nuclear marker, revealed exclusive cytoplasmic yellow fluorescence (**Figure 2C**), indicating that the VIP1-2 protein does not possess strong nuclear targeting capabilities. This result is consistent with deletion of the putative NLS from the VIP1-2 protein (Tsugama et al., 2012). Somewhat surprisingly, wild-type VIP1, when fused to GUS-Venus, also remains exclusively in the cytoplasm of both *Arabidopsis* and tobacco BY-2 protoplasts (**Figures 2D,E**). This result suggests either that the VIP1 nuclear localization signal sequence is not strong enough to target this large fusion protein to the nucleus, or that this fusion prevents phosphorylation of VIP1 serine-79 or some other aspect of VIP1 nuclear targeting.

### *vip1-2* Plants Show Altered Growth Characteristics

**FIGURE 3:**
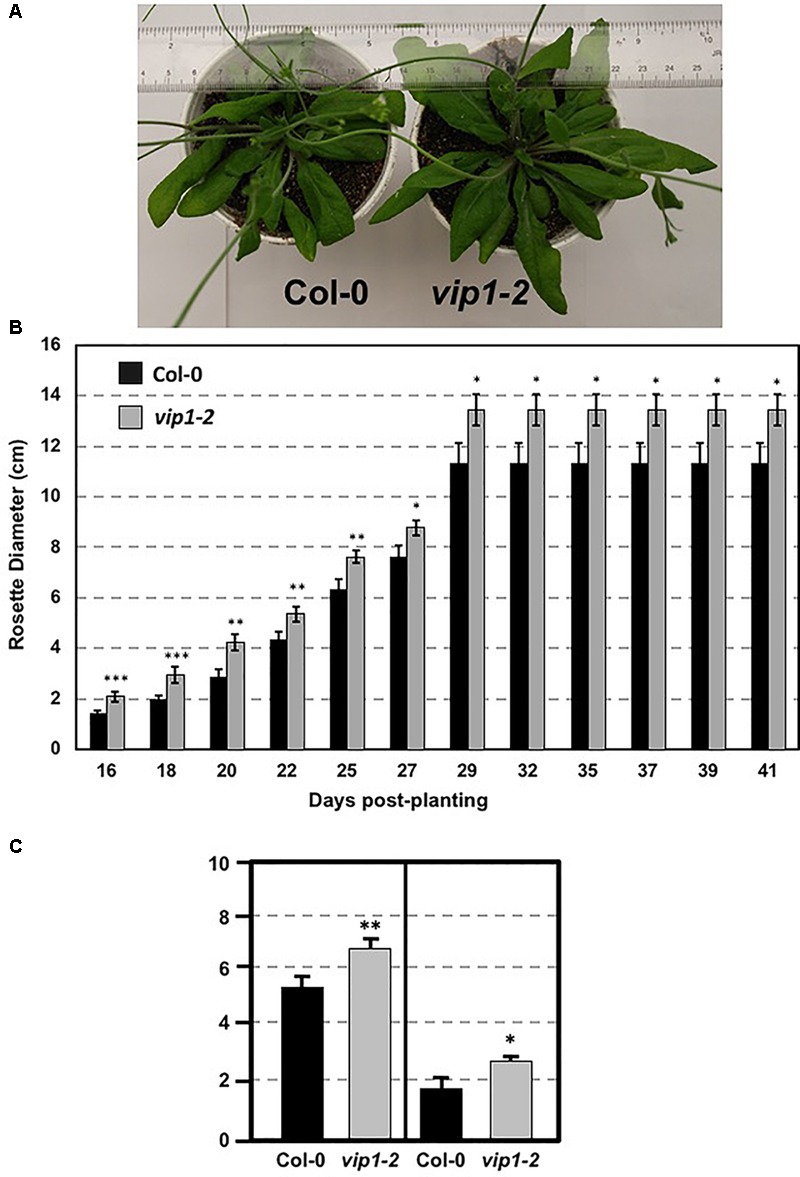
Growth of wild-type and *vip1-2* mutant *Arabidopsis* plants. **(A)** Mature (29-day-old) wild-type (Col-0, left) and *vip1-2* mutant (right) plants. **(B)** Bars represent the average diameter, ± SE, of leaf rosettes on 5–7 plants grown for the indicated number of days. **(C)** Bars represent the average leaf length (left) and width (right), ± SE, of the three largest leaves on five plants of each genotype grown for 29 days. Student’s *t*-test ^∗^*P*-value < 0.1, ^∗∗^*P*-value < 0.05, ^∗∗∗^*P*-value < 0.01.

We examined *vip1-2* plants for abnormal growth or developmental phenotypes. *vip1-2* plants exhibited increased rosette and leaf size compared to wild-type plants (**Figures 3A–C**). This growth phenotype suggests a role for *VIP1* in the regulation of rosette leaf development. However, flowering time did not differ significantly from that of wild-type plants (flowering occurred 26 days after seed sowing).

### *vip1-2* Plants Show Wild-Type Susceptibility to *Agrobacterium*-Mediated Transformation

**FIGURE 4:**
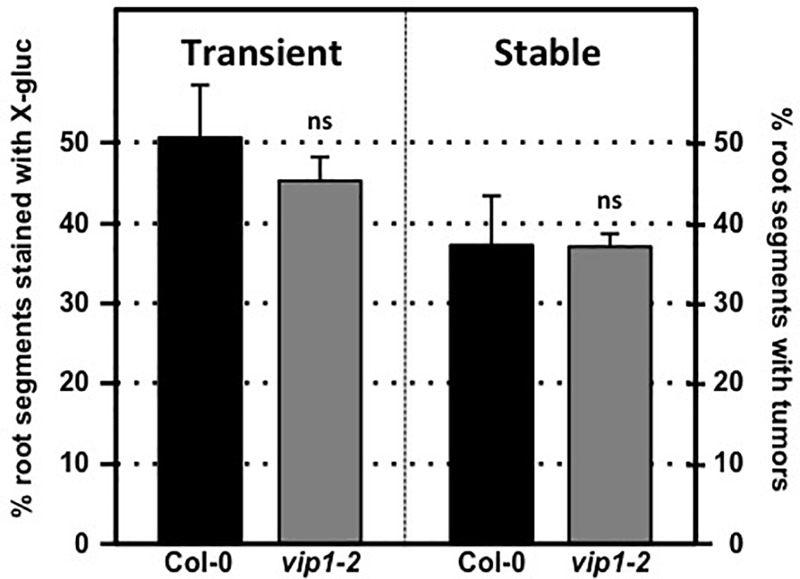
Transformation susceptibility of *Arabidopsis* wild-type and *vip1-2* mutant plants. *Agrobacterium*-mediated transient (left) or stable (right) transformation assays were conducted on wild-type and *vip1-2* mutant plants. Root segments were inoculated with 10^7^ cfu/ml of the *A. tumefaciens strains* At849 (transient) or A208 (stable). For the transient assay, the root segments were stained with X-gluc 6 days after infection. For stable transformation, tumors were scored 30 days after infection. Numbers represent an average of three biological replicates (each replicate containing > 60 root segments) ± SE. Student’s *t*-test. ns: not significant.

We tested transient and stable *Agrobacterium*-mediated transformation susceptibility of root segments from wild-type and *vip1-2* plants. Root segments were infected with a non-tumorigenic *Agrobacterium* strain carrying a GUS reporter, At849 (transient transformation), or the tumorigenic strain *A. tumefaciens* A208 (stable transformation; Nam et al., 1997, 1999; Zhu et al., 2003; Shi et al., 2014) at several bacterial concentrations. Root segments of wild-type and *vip1-2* plants had similar susceptibility to both transient and stable transformation at all bacterial concentrations tested (**Figure 4**; Supplementary Figure 2). These results correspond to our previous observations (Shi et al., 2014) that the *vip1-1* mutant is not deficient in transformation susceptibility.

### Individual *VIP1* Homologs Are Not Essential for *Agrobacterium*-Mediated Transformation

**FIGURE 5:**
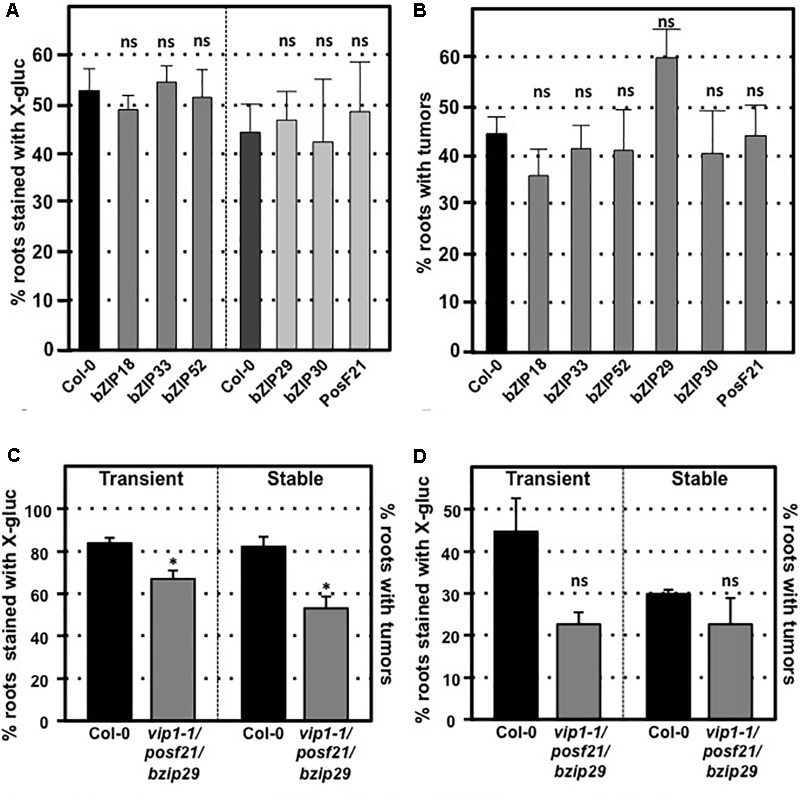
Transformation susceptibility of *Arabidopsis VIP1* homolog mutant roots. *Agrobacterium*-mediated transient or stable transformation assays were conducted on wild-type and VIP1 homolog mutant plants. Root segments of VIP1 homolog single gene mutants and one triple gene mutant were infected with *A. tumefaciens* At849 (transient) or A208 (stable) at the concentration of 10^6^ cfu/ml. For the transient assay, the root segments were stained with X-gluc 6 days after infection. For stable transformation, the tumors were scored 30 days after infection. Transient and stable transformation efficiencies of six VIP1 homolog mutants are shown in **(A,B)**, respectively. The transformation efficiencies of the triple gene mutant with an inoculum at 10^7^ cfu/ml and at 10^6^ cfu/ml are shown in **(C,D)**, respectively. Numbers represent an average of three biological replicates (each replicate containing > 60 root segments) ± SE. Student’s *t*-test ^∗^*P*-value < 0.05, ns: not significant.

*VIP1* is a single copy gene, but 11 close VIP1 homologs are present in *Arabidopsis*. VIP1 and these VIP1 homologs comprise the group I bZIP protein family. Their C-terminal regions, which include the bZIP domain, are highly similar to each other, whereas their N-terminal regions are variable (Jakoby et al., 2002; Tsugama et al., 2014). Of the 12 genes encoding the group I bZIP proteins, *VIP1*, and six other genes (*bZIP18, bZIP29, bZIP30, bZIP52, bZIP69*, and *PosF21*) are expressed at moderate levels in seedlings, roots, shoots, and flowers, whereas the other five genes (*UNE4, bZIP31, bZIP33, bZIP71*, and *bZIP74*) are hardly expressed in any of these tissues (Tsugama et al., 2014; Supplementary Figure 3). Many of these family members have similar subcellular localization, form homo- and heterodimers, and can similarly bind DNA fragments with the AGCTGT/G motif (Pitzschke et al., 2009; Tsugama et al., 2014, 2016; O’Malley et al., 2016). To test the importance of individual family members for transformation susceptibility, we obtained and confirmed homozygous mutants for six of the more highly expressed *VIP1* homologs (*bZIP18, bZIP29, bZIP30, bZIP33, bZIP52*, and *posF21*). No aberrant phenotypes were observed in these single knockout mutants under normal growth conditions. Transient and stable root transformation assays indicated that each mutant had transformation susceptibility similar to that of wild-type plants (**Figures 5A,B**). Thus, in addition to VIP1, none of these six transcription factors is essential for *Agrobacterium*-mediated transformation. We additionally tested a triple mutant (*vip1-1*/*posf21*/*bzip29*) for transient and stable transformation susceptibility. Using two concentrations of bacterial inoculum, the *vip1-1*/*posf21*/*bzip29* mutant had a slight (1.5-fold) reduction in both transient and stable transformation efficiency (**Figures 5C,D**).

### Dominant Repression of VIP1 Family Function by a VIP1-SRDX Fusion Does Not Affect Transformation Susceptibility

**FIGURE 6:**
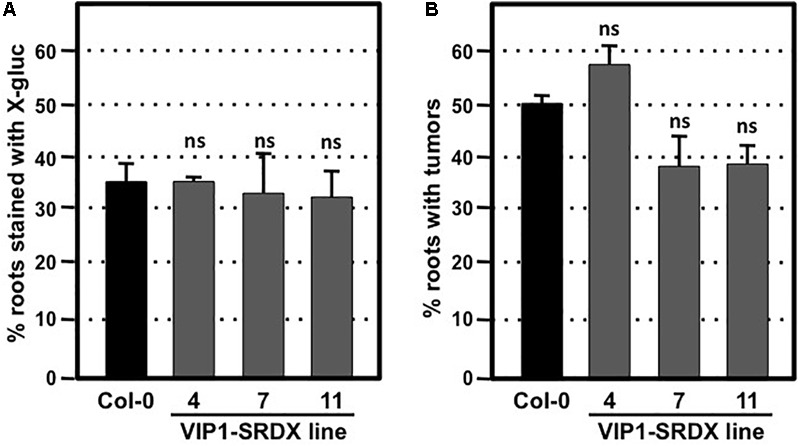
Transformation susceptibility of *Arabidopsis* wild-type and VIP1-SRDX mutant roots. *Agrobacterium*-mediated transient or stable transformation assays were conducted on wild-type and VIP1-SRDX plants. Root segments were inoculated with the strains *A. tumefaciens* At849 (10^6^ cfu/ml for transient) or A208 (10^7^ cfu/ml for stable). For transient transformation **(A)**, root segments were stained with X-gluc 6 days after infection; for stable transformation **(B)**, tumors were scored 30 days after infection. Numbers represent an average of three biological replicates (each replicate containing > 60 root segments) ± SE. Student’s *t*-test. ns: not significant.

To circumvent potential redundant roles among VIP1 family members, we assayed the transformation susceptibility of root segments from three transgenic *Arabidopsis* lines expressing VIP1 fused to the EAR motif repression domain SRDX (Mitsuda et al., 2006; Tsugama et al., 2016). The three independent lines of VIP1-SRDX plants used in this study all showed high expression levels of *VIP1-SRDX* and root waving phenotypes in a previous study (Tsugama et al., 2016), indicating the efficacy of the EAR motif in repressing expression of genes regulated by VIP1 family members. However, they showed transient and stable transformation susceptibility similar to that of wild-type plants (**Figures 6A,B**). Tumor size and morphology also did not change on roots of these lines. We conclude that VIP1 and its homologs are not essential for *Agrobacterium*-mediated transformation.

### Subcellular Localization of VIP1 Homologs and Their Interactions With VirE2

**FIGURE 7:**
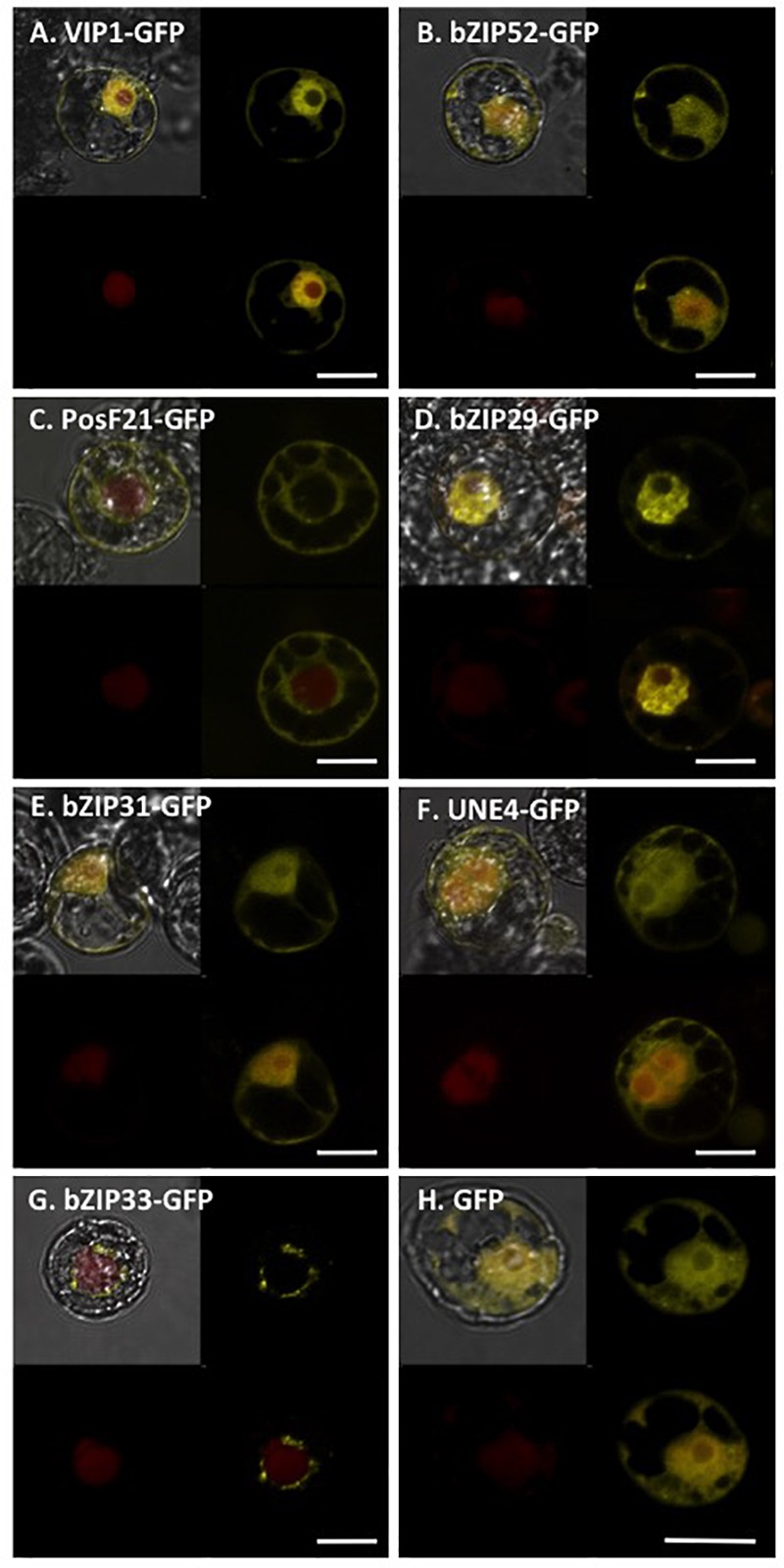
Subcellular localization of VIP1 and its homologs in tobacco BY-2 protoplasts. DNA of Venus-tagged VIP1 or its homologs were co-transfected with a nuclear marker mRFP-NLS into tobacco BY-2 protoplasts. Cells were imaged by confocal microscopy 16 h after transfection. Four images of each cell are presented (clockwise from top left: merged YFP, mRFP, and DIC; YFP; YFP + mRFP; mRFP). Bars indicate 20 μm.

We transfected tobacco BY-2 protoplasts using constructs encoding GFP-tagged VIP1, bZIP52, PosF21, bZIP29, bZIP31, UNE4, and bZIP33 expressed from a Cauliflower Mosaic Virus (CaMV) 35S promoter (**Figure 7**). The subcellular localization of VIP1, bZIP52, bZIP31, and UNE4 is in both the cytoplasm and the nucleus, except that VIP1 localizes predominantly to the nucleoplasm (**Figure 7A**), whereas the other transcription factors also localized to the nucleolus (**Figures 7B,E,F**). PosF21 and bZIP33 localized predominantly to the cytoplasm (**Figures 7C,G**), with bZIP33 showing perinuclear aggregates (**Figure 7G**). bZIP29 showed exclusively nucleoplasmic localization (**Figure 7D**). Free GFP localized throughout the cytoplasm and nucleus (**Figure 7H**). Thus, although these related transcription factors showed overlapping subcellular localization patterns, none of these patterns is identical to that of VIP1.

**FIGURE 8:**
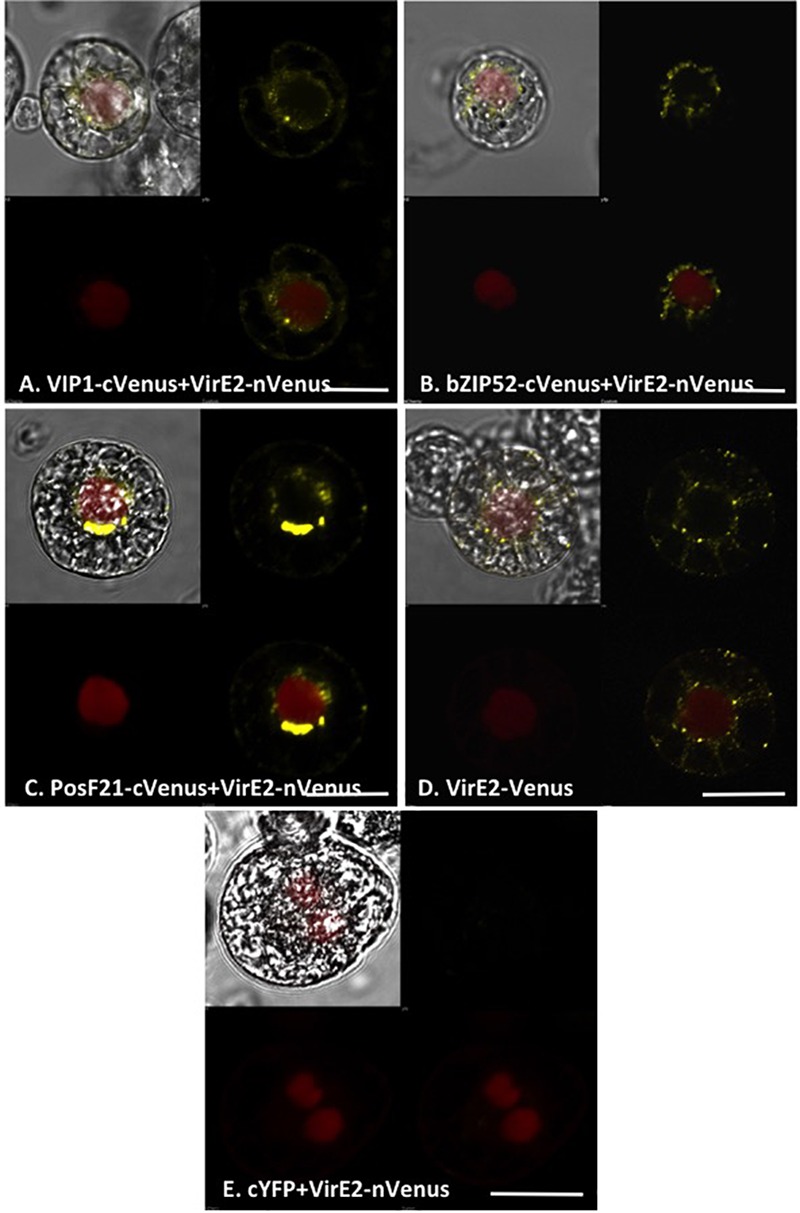
Subcellular localization of complexes formed by VirE2 with VIP1 homologs in tobacco BY-2 protoplasts. Tobacco BY-2 protoplasts were co-transfected with constructs comprised of the indicated cVenus-tagged VIP1 homologs and VirE2-nVenus **(A–C)**; a construct encoding VirE2-Venus **(D)**, or constructs encoding VirE2-nVenus and cCFP **(E)**. A nuclear marker encoding mRFP-NLS was also included in all transfection experiments. The cells were imaged by confocal microscopy after 16 h. Four images of each cell are presented (clockwise from top left: merged YFP, mRFP, and DIC; YFP; YFP + mRFP; mRFP). Bars indicate 20 μm.

We examined the interaction of VirE2 with VIP1, bZIP52, and PosF21 using BiFC. VIP1-VirE2 complexes localized to the perinuclear area and formed aggregates (**Figure 8A**; Shi et al., 2014). The interaction and co-localization patterns of bZIP52 and PosF21 with VirE2 (**Figures 8B,C**) resemble the pattern of VirE2 localization (**Figure 8D**). These data suggest that through interaction, VirE2 relocalizes these transcription factors in plant cells (compare **Figures 7, 8**). In our control, we did not detect interaction of VirE2-nYFP with cCFP (**Figure 8E**).

### VIP1 Target Gene Expression in the Absence and Presence of *Agrobacterium*

**FIGURE 9:**
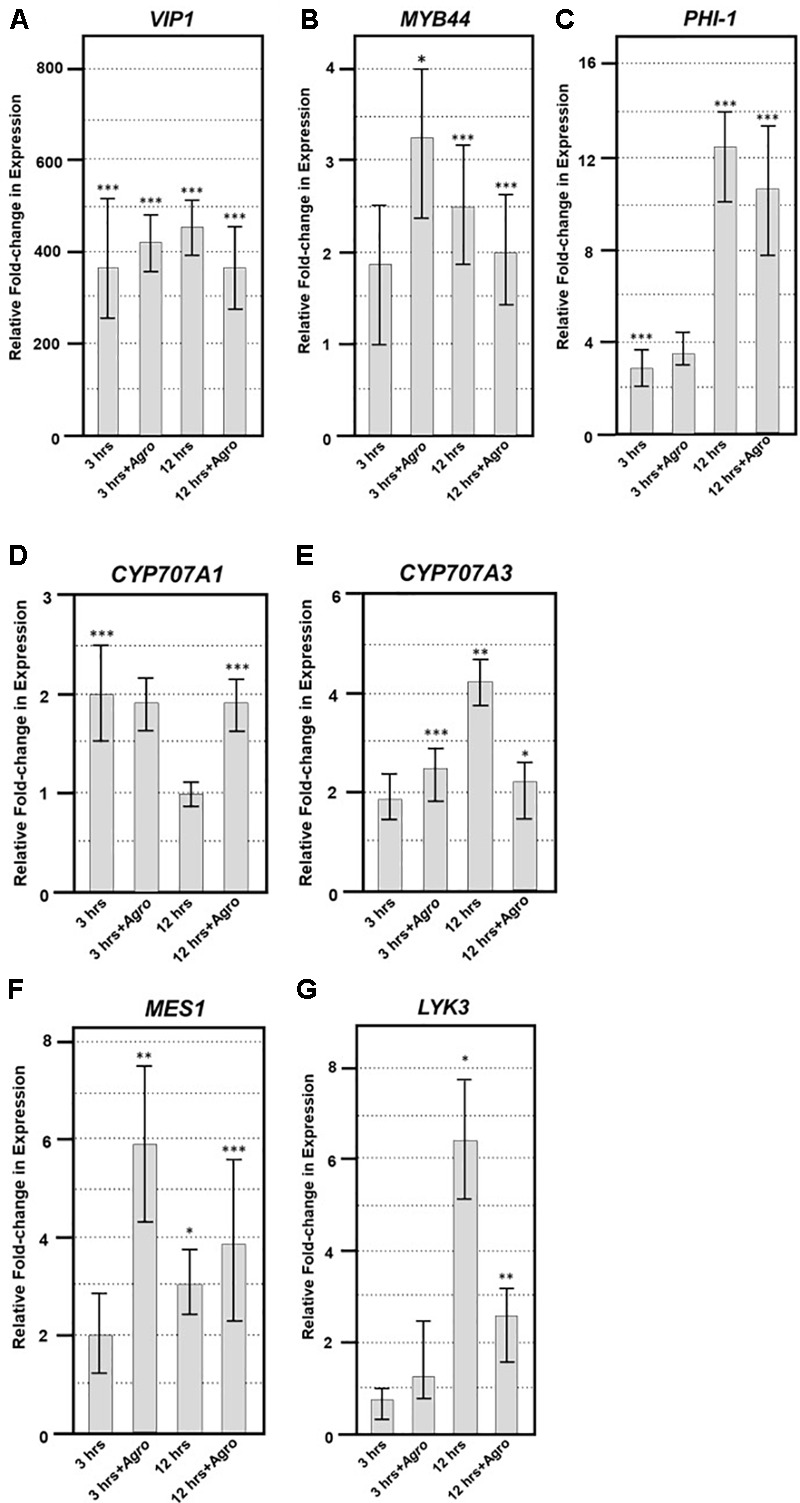
Quantitative RT-PCR of VIP1 target and fungal defense genes. Quantitative RT-PCR of **(A)** *VIP1* transgene, **(B)** *MYB44*, **(C)** *PHI-1*, **(D)** *CYP707A1*, **(E)** *CYP707A3*, **(F)** *MES1*, and **(G)** *LYK3* gene expression in induced relative to that of non-induced roots (*Y*-axis). Results represent an average of three replicates ± SE. Relative expression is shown after 3 and 12 h of induction in the absence or presence of *Agrobacterium* (+Agro) on the *X*-axis. Asterisks indicate SE according to Student’s *t*-test: ^∗^*P*-value < 0.05, ^∗∗^*P*-value < 0.01, ^∗∗∗^*P*-value < 0.001.

**FIGURE 10:**
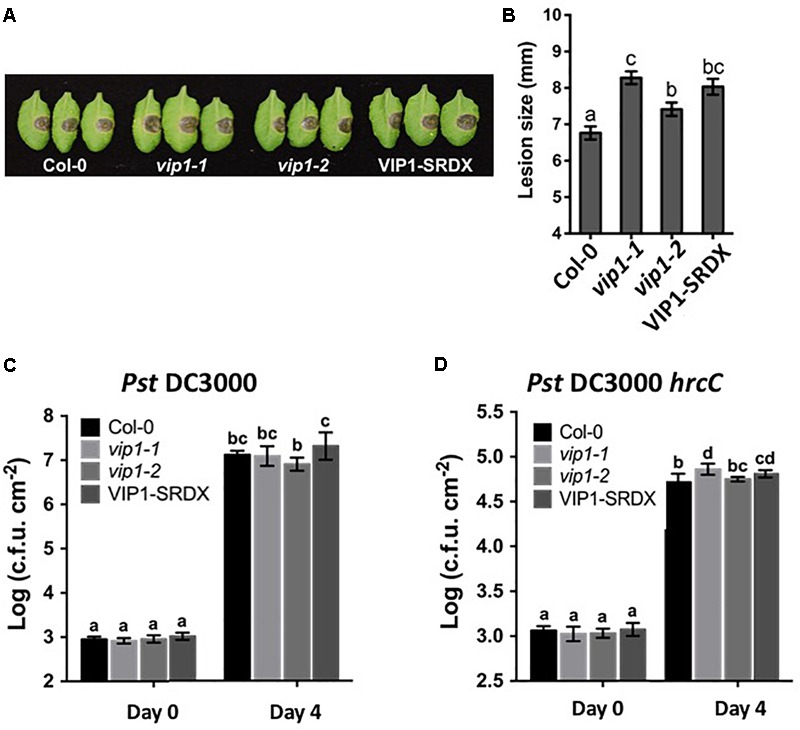
VIP1 is important for fungal but not bacterial infection of *Arabidopsis*. **(A)** Disease symptoms on leaves of various plants 3 days after inoculation with 5 μl of *B. cinerea* at the concentration *of* 1.0 × 10^5^ spores/ml; **(B)** Average lesion size of 18 leaves of each genotype (36 leaves for Col-0) after *B. cinerea* inoculation; **(C)** Leaves of 5-week-old plants were syringe inoculated with *Pseudomonas syringe* c.v. *tomato Pst* DC3000 (A_600_ = 0.001) or **(D)** *Pst* DC3000 *hrcC* (A_600_ = 0.005). Growth of *bacteria on leaves* was measured at 0 and 4 dpi. Average numbers of bacteria and SDs were obtained from three replicates, each consisting of six leaf disks. Different letters indicate significant differences (*P* < 0.05, Student’s *t*-test).

To elucidate the expression of VIP1 target genes in the presence of *Agrobacterium*, we generated transgenic *A. thaliana* expressing *VIP1* under the control of an inducible promoter. We incubated roots of these plants in induction or control solutions for 0, 3, or 12 h in the absence or presence of the avirulent strain *A. tumefaciens* A136 lacking a Ti plasmid. Incubation with bacteria induces plant PAMP (pattern associated molecular pattern) defense responses. After various times, we harvested root tissue and isolated total RNA. We performed quantitative RT-PCR analysis to measure the expression of previously identified VIP1 target genes (Pitzschke et al., 2009; Tsugama et al., 2012, 2014; Andrea Pitzschke, personal communication). These experiments were performed as three technical replicates each of three biological replicates. Representative data are shown in **Figures 9A–E**, and the full analysis is shown in Supplementary Table 4. The *VIP1* transgene was strongly expressed in the induced but not the non-induced samples, both in the absence and in the presence of *Agrobacterium* (**Figure 9A**). The VIP1 target gene *MYB44* (*At5g67300*) showed slightly elevated expression to similar levels in all of the induced samples compared to the non-induced samples, both in the presence and in the absence of *Agrobacterium* (**Figure 9B**). The putative VIP1 target gene *PHI-1* (*At1g35140*) showed the highest expression 12 h after induction in the presence of *Agrobacterium* (**Figure 9C**). Although *CYP707A1* (*At4g19230*) showed a modest two-fold response to induction of *VIP1, CYP707A3* (*At5g45340*) showed an even greater increase in expression, especially 12 h after induction in the presence of *Agrobacterium*. However, the expression of *CYP707A3* in the presence of *Agrobacterium* was at a level similar to that found in the 3-h samples (**Figure 9E**). *CYP707A3* is involved in the inactivation of ABA signaling, suggesting that VIP1 may play a role in modulating ABA responses during stress responses (Tsugama et al., 2012). ABA is a key hormone involved in defense responses against fungal pathogens such as *Botrytis cinerea* (Audenaert et al., 2002; Fan et al., 2009; Sivakumaran et al., 2016). Therefore, we tested the susceptibility of *vip1* mutant plants to *Botrytis cinerea* (**Figure 10**).

### *vip1* Mutant and *VIP1-SRDX* Lines Show Increased Susceptibility to *Botrytis cinerea*, but Not to *Pseudomonas syringae* Infection

VIP1 is a target of the MAPK cascade and has been proposed to be involved in defense responses (Pitzschke et al., 2009). Although we were unable to find a role for VIP1 and its homologs in defense against *Agrobacterium*, we considered that VIP1 may play a role in defense against other pathogens. We therefore conducted pathogenesis assays, using *Pseudomonas syringae* and *Botrytis cinerea*, on wild-type, *vip1-1, vip1-2*, and *VIP1-SRDX* plants. Leaf lesion size was significantly larger on *B. cinerea* infected *vip1-1, vip1-2*, and *VIP1-SRDX* leaves than on wild-type leaves, indicating that VIP1, and perhaps additionally its paralogs, are involved in defense against *Botrytis* infection (**Figures 10A,B**).

The *vip1* mutants and *VIP1-SRDX* lines responded similarly as did wild-type plants to treatment with both a virulent (*Pst* DC3000) and avirulent (*Pst* DC3000 *hrcC*) *P. syringae* strains (**Figures 10C,D**). These results suggest that *VIP1* plays a role in *B. cinerea*, but not *P. syringae* and *A. tumefaciens*, defense. We also found that expression of the fungal defense genes *MES1* (*At2g23620*) and *LYK3* (*At1g51940*) were elevated after induction of *VIP1* transgene expression, suggesting a role for VIP1 in fungal defense (**Figures 9F,G**; Vlot et al., 2008; Paparella et al., 2014).

### *vip1* Mutant and *VIP1-SRDX* Lines Are Sensitive to Exogenous ABA During, but Not After, Germination

**FIGURE 11:**
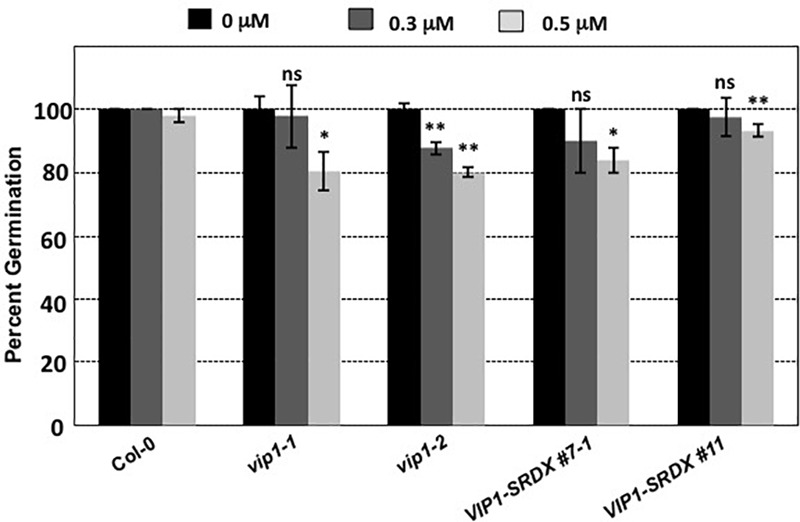
Germination of wild-type, *vip1* mutants, and a VIP1-SRDX line on medium containing ABA. Seeds of the indicated lines were germinated on B5 medium containing 0, 0.3, or 0.5 μM ABA. Data represent the average percent germination ± SE. Student’s *t*-test ^∗^*P*-value < 0.1, ^∗∗^*P*-value < 0.05, ns: not significant.

*Botrytis cinerea* produces exogenous ABA to suppress plant defense responses (Audenaert et al., 2002; Fan et al., 2009; Sivakumaran et al., 2016). *VIP1* may play a role in ABA signaling (Tsugama et al., 2012, 2013, 2014). Under hypo-osmotic conditions, VIP1 re-localizes to the nucleus and activates transcription of *CYP707A1* and *CYP707A3* (Tsugama et al., 2012) which encode proteins that degrade ABA and are therefore involved in osmosensory regulation of plant growth (Kushiro et al., 2004; Umezawa et al., 2006; Supplementary Figure 4). In the absence of *VIP1*, plants may be less able to degrade exogenous ABA, which may explain the increased susceptibility of *vip1* mutant plants and *VIP1-SRDX* lines to *B. cinerea* infection. Because ABA is also a negative regulator of germination (Gimeno-Gilles et al., 2009; Supplementary Figure 4), we hypothesized that *vip1* mutant and *VIP1-SRDX* lines may display altered germination in the presence of exogenous ABA. We germinated seeds of wild-type, *vip1-1, vip1-2*, and *VIP1-SRDX* lines 7–1 and 11 on medium containing either 0, 0.3, or 0.5 μM ABA. In the presence of ABA, almost all the wild-type seeds germinated within 8 days after imbibition. Seeds of the *vip1-1* and *vip1-2* mutants, and two *VIP1-SRDX* lines, showed reduced germination in the presence of ABA (**Figure 11**).

Low concentrations of ABA promote root growth, whereas high concentrations inhibit growth (Pilet and Saugy, 1987; Sharp and LeNoble, 2002). To elucidate whether *VIP1* plays a role in ABA signaling during root growth, we first germinated *vip1* mutant and *VIP1-SRDX* plants on MS medium, then transferred the seedlings to plates containing 0, 2, or 20 μM ABA to continue growth. The rate of root growth did not significantly differ from that of wild-type for any of the *vip1* mutant or *VIP1-SRDX* lines (Supplementary Figure 5). These results suggest that although *VIP1* appears important for ABA defense signaling and germination, it does not play a role in ABA-dependent regulation of root growth.

### *vip1* Mutant and *VIP1-SRDX* Roots Are More Tolerant to Growth in High Salt

**FIGURE 12:**
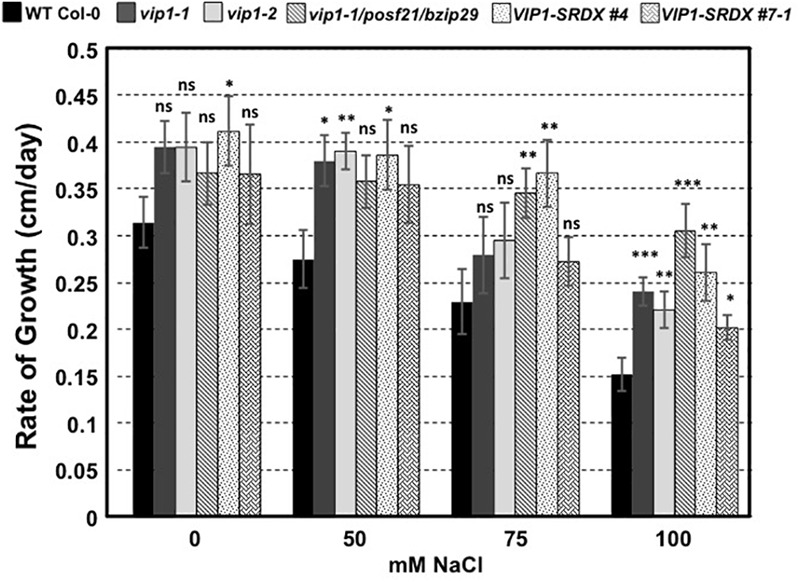
Root growth rates of wild-type, *vip1* mutant, and VIP1-SRDX lines on various concentrations of NaCl. Data represent the average rate of root growth ± SE. Student’s *t*-test ^∗^*P*-value < 0.05, ^∗∗^*P*-value < 0.01, ^∗∗∗^*P*-value < 0.001, ns: not significant.

To determine whether VIP1 also plays a role under hyper-osmotic conditions, we performed seed germination and root growth assays on wild-type Col-0, *vip1-2, vip1-1*/*posf21*/*bzip29* mutants, and VIP1-SRDX lines. All seeds germinated well on medium containing elevated concentrations of NaCl (Supplementary Figure 6). However, roots of all *vip1* mutant lines, and two *VIP1-SRDX* lines, grew better on medium containing salt than did wild-type roots (**Figure 12**). These results indicate that *VIP1* plays a role in root growth under salt stress conditions.

## Discussion

Previous reports indicated that, compared to wild-type plants, *vip1-1 A. thaliana* and *VIP1* antisense tobacco plants showed reduced stable transformation susceptibility, suggesting an important role for *VIP1* in *Agrobacterium*-mediated transformation (Tzfira et al., 2001, 2002; Li et al., 2005). As a consequence of these reports, and observations that plant-generated VirE2 localizes to the nucleus (Zupan et al., 1996) but cannot interact with importin α-1 (AtKapα; Ballas and Citovsky, 1997), VIP1 was proposed to act as an adaptor molecule between importin α-1 and VirE2 for nuclear entry of VirE2-bound T-DNA (the Trojan-horse model; Djamei et al., 2007). However, we have observed that VirE2 can interact with all tested *Arabidopsis* importin α isoforms *in vitro*, in yeast, and in plants (Bhattacharjee et al., 2008). We and others have observed that VirE2 and VIP1-VirE2 complexes synthesized *in planta* localize to the cytoplasm (Bhattacharjee et al., 2008; Lee et al., 2008; Sakalis et al., 2013; Shi et al., 2014; this study), indicating that VIP1 does not act as an adaptor to localize VirE2 to the nucleus. Recent reports, however, suggest that some VirE2 molecules delivered from *Agrobacterium* may reach the nucleus (Li et al., 2014; Yang et al., 2017). Thus, the role of VirE2 in helping deliver T-strands to the plant nucleus remains controversial. Regardless of the role of VirE2 in nuclear import of T-strands, this current, and a previous, study (Shi et al., 2014) found no significant change in *Agrobacterium*-mediated transformation susceptibility in any *VIP1* mutant background or in *VIP1* overexpressing transgenic lines. Therefore, our data do not support the Trojan Horse model (Djamei et al., 2007).

Although we could not find a role for VIP1 in *Agrobacterium*-mediated plant transformation, we were concerned that other group I bZIP transcription factors related to *Arabidopsis* VIP1 could mask the effect of VIP1 on transformation. *VIP1* is one of a 12-gene family whose members may have redundant functions. Analysis of null mutants of six individual *VIP1* homologs did not reveal any transformation phenotypes, and the *vip1-1*/*posf21*/*bzip29* triple mutant showed only a modest reduction in transformation susceptibility, suggesting that some VIP1 family members may slightly potentiate transformation. We therefore analyzed transgenic lines overexpressing VIP1 fused to a SRDX repression domain. The binding of other transcription factors to the promoters of *VIP1* target genes is blocked in these lines (Mitsuda et al., 2006; Tsugama et al., 2016). *VIP1-SRDX* lines also displayed transformation characteristics similar to those of wild-type plants. We therefore conclude that full expression of *VIP1* and its homologs is not required for *Agrobacterium*-mediated transformation. It is possible, however, that residual expression of VIP1 target genes may facilitate transformation.

While testing inducible VIP1 plants in the absence and presence of *Agrobacterium*, we observed differential expression of the VIP1 target genes *MYB44* (*At5g67300*) and *CYP707A3* (*At5g45340*) previously identified in the literature (**Figures 9B,E**; Pitzschke et al., 2009; Tsugama et al., 2012, 2014). Pitzschke et al. (2009) found that *MYB44* was upregulated in wild-type plants after treatment with flg22, and that the gene contains multiple copies of a VIP1 responsive element (VRE) in its promoter. We were also able to detect upregulation of the putative VIP1 target gene *PHI-1* (*At1g35140*) in response to VIP1 induction (**Figure 9C**; Andrea Pitzschke, personal communication). *CYP707A3* expression also increases upon *VIP1* induction, as well as after tissue rehydration and in the presence of mannitol in *VIP1* overexpressing plants (**Figure 9E**; Tsugama et al., 2012). Although Tsugama et al. (2012, 2014) showed that *CYP707A1* (*At4g19230*) is differentially expressed under the same conditions as is *CYP707A3*, we only detected a modest change in *CYP707A1* expression in our experiments (**Figure 9D**), suggesting that *CYP707A1* upregulation requires conditions not present in our protocol.

VIP1 is a phosphorylation target of MPK3 and has been proposed to be involved in plant defense responses (Pitzschke et al., 2009). Increased susceptibility of *vip1-1, vip1-2*, and *VIP1-SRDX A. thaliana* plants to *Botrytis cinerea*, but not to *P. syringae*, suggests that *VIP1* may play a role in fungal but not bacterial defense responses. Resistance to broad host necrotrophic fungi such as *B. cinerea* is mediated by quantitative resistance mechanisms involving the contributions of many genes (Poland et al., 2009; Lai and Mengiste, 2013). The *VIP1* gene contributes to this resistance, as indicated by the increase in disease lesion size when *VIP1* is debilitated (**Figure 10A**). This model is supported by our observation that the *LYK3* gene, involved in response to chitin, is upregulated in inducible *VIP1* plants (**Figure 9G**). VIP1 may be phosphorylated by MPK3 in response to *B. cinerea*, leading to the activation of its target genes *CYP707A1* and *CYP707A3*, which are ABA degradation enzymes (Kushiro et al., 2004; Umezawa et al., 2006). *CYP707A1* and *CYP707A3* may be important for the degradation of exogenous ABA produced by *B. cinerea*, preventing the suppression of defense responses (Audenaert et al., 2002; Fan et al., 2009; Sivakumaran et al., 2016). The role of ABA in fungal infection is consistent with the observation that ABA-deficient tomato plants are highly resistant to *B. cinerea* infection (Asselbergh et al., 2007). The precise role of *VIP1* in defense signaling during *B. cinerea* infection remains unknown. Measuring the expression of *VIP1* target genes throughout infection should provide clues as to how *VIP1* contributes to defense against *B. cinerea* during early and late stages of infection.

The altered growth of *vip1-2* leaves (**Figures 3A,B**) suggests a role for VIP1 in plant growth and development. This role is supported by the observation of increased touch-induced root waving in *VIP1-SRDX* plants (Tsugama et al., 2016) and is consistent with previous findings that two other group I bZIP proteins, bZIP29 and bZIP30, also regulate leaf growth (Lozano-Sotomayor et al., 2016; Van Leene et al., 2016). This and previous studies also suggest a role for VIP1 in the regulation of abiotic stress responses, specifically to hypo- and hyperosmotic conditions (**Figure 12**; Tsugama et al., 2012). A previous study did not observe any major growth differences of *VIP1-SRDX* compared to wild-type plants in the presence of ABA or under hyperosmotic conditions (mannitol) after 3 weeks of growth (Tsugama et al., 2016). Our study, however, measured seed germination and the rate of root growth of plants at earlier times (less than 12 days after plating). This assay allowed us to quantify better the sensitivity of these plants to ABA and hyperosmotic conditions. VIP1 enters the nucleus and binds to the promoters of its target genes, *CYP707A1* and *CYP707A3*, upon rehydration of plant roots, leading to an increase in their expression (Tsugama et al., 2012). It is unknown whether the increase in the expression of *CYP707A1* and *CYP707A3* by VIP1 after rehydration leads to the degradation of ABA or contributes to some other signaling pathway. Whether VIP1 affects plant growth and development under various osmotic conditions via *CYP707A1*/*CYP707A3* degradation of ABA or by other ABA-dependent or -independent signaling mechanisms will require further investigation.

## Author Contributions

RL and SG designed the experiments. RL, L-YL, SL, and SG conducted the experiments. DT contributed to the reagents. RL and SG wrote the article with contributions from L-YL, TM, and DT.

## Conflict of Interest Statement

The authors declare that the research was conducted in the absence of any commercial or financial relationships that could be construed as a potential conflict of interest.

## Footnotes

1 http://www.gene-quantification.de/rest-2009.html

2 https://www.thermofisher.com

3 http://www.gene-quantification.de/rest-2009.html
