# Supplementary material for: VIP1 and Its Homologs Are Not Required for Agrobacterium-Mediated Transformation, but Play a Role in Botrytis and Salt Stress Responses
Source: Front Plant Sci. 2018 Jun 12;9:749. doi: 10.3389/fpls.2018.00749 (PMC6005860; doi:10.3389/fpls.2018.00749)
Supplement: Supplementary file 1 [file Table_1.docx]

**Supplemental Table 1. Bacterial strains used in this study**

| **Strain name** | **Description** | **Antibiotic resistance^a^** | **Reference** |
| --- | --- | --- | --- |
|  | ***E. coli* strains** |  |  |
| psgR-Cas9-At | sgRNA scaffold plus Cas9 | Amp | Feng et al., 2013 |
| VIP1-403 | sgRNA construct #403 (GTTCAGGAGAGAAGAAGAAA) in psgR-Cas9-At | Amp | This study |
| VIP1-504 | sgRNA construct #504 (CGCTCTGAAACCTTCTTCTC) | Amp | This study |
| VIP1-506 | sgRNA construct #506 (TTCTCCCATGTCCGTTGATT) | Amp | This study |
| DH10B | F^-^ [mcrA](http://ecoliwiki.net/colipedia/index.php/mcrA) Δ(*mrr*-*hsd*RMS-*mcr*BC) Φ80d*lac*ZΔM15 Δ*lac*X74 *end*A1 *rec*A1 *deo*R Δ(*ara*,*leu*)7697 *ara*D139 *gal*U *gal*K *nup*G *rps*L λ^-^ | None | Durfee et al., 2008 |
| pCAMBIA1300 | Binary sequencing vector | Kan | Hajdukiewicz et al., 1994 |
| E4351 | T-DNA binary vector VIP1-CRISPR sgRNA #403-Cas9 | Kan | This study |
| E4352 | T-DNA binary vector VIP1-CRISPR sgRNA #504-Cas9 | Kan | This study |
| E4353 | T-DNA binary vector VIP1-CRISPR sgRNA #506-Cas9 | Kan | This study |
| E886 | pBluescript (pBS) II KS (+) in DH5α | Amp | Stratagene |
| E4443 | pBS-vip1-2 (full-length cDNA) | Amp | This study |
| E3857 | pSAT4A-VIP1-Venus | Amp | Shi et al., 2014 |
| E4451 | pSAT4A-vip1-2 (full-length cDNA)-Venus | Amp | This study |
| E3170 | pSAT6-P_35S_-mRFP-NLS | Amp | Citovsky et al., 2006 |
| E4516 | pBS-vip1-2peptide | Amp | This study |
| E3835 | pSAT4A-GUS-Venus-N | Amp | Lee et al., 2008 |
| E4521 | pSAT4A-vip1-2peptide-GUS-Venus | Amp | This study |
| E4517 | pSAT4A-VIP1-GUS-Venus | Amp | This study |
| E3232 | pSAT4A-nVenus-N | Amp | Lee et al., 2008 |
| E4518 | pSAT4A-vip1-2peptide-nVenus | Amp | This study |
| E3347 | pSAT4A-cCFP-N | Amp | Lee et al., 2008 |
| E4522 | pSAT4A-vip1-2peptide-cCFP | Amp | This study |
| E3680 | Pnos-VirE2(pTiA6 from A348)-nVenus | Amp | Lee et al., 2008; Sciaky et al., 1978 |
| E4462 | pBS-P_35S_-VIP1-GFP | Amp | Tsugama laboratory |
| E4463 | pBS-P_35S_-bZIP52-GFP | Amp | Tsugama laboratory |
| E4464 | pBS-P_35S_-PosF21-GFP | Amp | Tsugama laboratory |
| E4465 | pBS-P_35S_-bZIP29-GFP | Amp | Tsugama laboratory |
| E4466 | pBS-P_35S_-bZIP31-GFP | Amp | Tsugama laboratory |
| E4468 | pBS-P_35S_-bZIP33-GFP | Amp | Tsugama laboratory |
| E4469 | pBS-P_35S_-MCS-GFP(empty vector control) | Amp | Tsugama laboratory |
| E4470 | pBS-P_35S_-VIP1-VC80 | Amp | Tsugama laboratory |
| E4471 | pBS-P_35S_-bZIP52-VC80 | Amp | Tsugama laboratory |
| E4472 | pBS-P_35S_-PosF21-VC80 | Amp | Tsugama laboratory |
| E4473 | pBS-P_35S_-MCS-VC80 (empty vector control) | Amp | Tsugama laboratory |
| E4132 | pSAT4-P_35S_-VIP1 | Amp | Lee et al., 2008 |
| E4224 | pSAT1-Inducible Promoter (minimal 35S-LexA operator) | Amp | Lee et al., 2008 |
| E4275 | pSAT1-pI-VIP1 | Amp | This study |
| pE4280 | T-DNA binary vector XVE | Spec | This study |
| pE4288 | T-DNA binary vector inducible VIP1-XVE | Spec | This study |
|  | ***Agrobacterium* strains** |  |  |
| A208 | Tumorigenic; pTiT37 in A136 | Rif | Sciaky et al., 1978 |
| GV3101 | Non-tumorigenic, disarmed pTiC58 in C58 background | Rif, Gent | Koncz and Schell, 1986 |
| At2 | Non-tumorigenic; A136 | Rif | Sciaky et al., 1978 |
| At849 | pBISN1 in GV3101 | Rif, Gent, Kan | Narasimhulu et al., 1996 |
| At2115 | pE4351 in GV3101 | Rif, Gent, Spec | This study |
| At2116 | pE4352 in GV3101 | Rif, Gent, Spec | This study |
| At2117 | pE4353 in GV3101 | Rif, Gent, Spec | This study |
| At2082 | pE4288 in GV3101 | Rif, Gent, Spec | This study |

^a^Amp, ampicillin; Gent, gentamicin; Kan, kanamycin; Rif, rifampicin; Spec, spectinomycin
