# Supplementary material for: VIP1 and Its Homologs Are Not Required for Agrobacterium-Mediated Transformation, but Play a Role in Botrytis and Salt Stress Responses
Source: Front Plant Sci. 2018 Jun 12;9:749. doi: 10.3389/fpls.2018.00749 (PMC6005860; doi:10.3389/fpls.2018.00749)
Supplement: Supplementary file 2 [file Table_2.docx]

**Supplemental Table 2. Primer sequences used in this study**

| **Primer Name** | **Sequence (5’ to 3’)** | **Purpose** |
| --- | --- | --- |
| VIP1-BglII-FP1 | CGAGATCTATGGAAGGAGGAGGAAG | Screening for *vip1* mutant and cloning of *vip1-2* transcript |
| VIP1-BamHI-RP1 | AAGGATCCCGCCTCTCTTGGTGAAAT | Cloning of *vip1-2* transcript |
| *vip1-2* peptide primer | TTGGATCCTGATGATGATTCCCTTTTCTTC | Cloning of *vip1-2-*GUS-Venus fusion construct |
| eVIP1 qPCR Fwd | CATCTCAGCAATCGGCAATGAATCAG | Real-time PCR |
| eVIP1qPCR Rev | TCAGCAAACATATATAGGTGACACGAACTC | Real-time PCR |
| MYB44 (AT5G67300) RT-PCR Fwd | GTTAAGAGATCGGTGAGTGCG | Real-time PCR |
| MYB44 (AT5G67300) RT-PCR Rev | GGTGGATCATCGGAAGAAGAC | Real-time PCR |
| PHI-1 (AT1G35140)  qPCR Fwd | CAACGAACCCGTTCGGAGATG | Real-time PCR |
| PHI-1 (AT1G35140)  qPCR Rev | TCGTTGCATCCACAAGTAACTCTCC | Real-time PCR |
| VIP1-Transgene (AT1G43700) qPCR Rev | AGACTGGTGATTTTTGCGGACTCTAG | Real-time PCR |
| CYP707A1 (AT4G19230) -Fwd | TGGAACCCACTCGTGTCCTGGA | Real-time PCR |
| CYP707A1 (AT4G19230) -Rev | CCCGTCGCTCGCTCCAACAA | Real-time PCR |
| CYP707A3 (AT5G45340) -Fwd | TCGAAGTTGCGCCGAAACCGA | Real-time PCR |
| CYP707A3 (AT5G45340) -Rev | GGCCCTACGATTGACCATCTGTACT | Real-time PCR |
| MES1 (AT2G23620) qFwd | CAGAAGAACGCCAGAGATGGATGATTG | Real-time PCR |
| MES1 (AT2G23620) qRev | TTGTCCGCGATTTTCAGGAAGTAATCAC | Real-time PCR |
| LYK3 (AT1G51940) qFwd | ATCCACCAGCTCCTTCTCCT | Real-time PCR |
| LYK3 (AT1G51940) qRev | TGCAAGCACAACTCCAAGAC | Real-time PCR |
| Left Border Primer (SALK) | TGGTTCACGTAGTGGGCCATCG | T-DNA insertion line genotyping |
| bZIP30 LP | CCCCATTTCAGGTCAAATTTC | T-DNA insertion line genotyping |
| bZIP30 RP | AATTCCCTTCACCTGAATTGG | T-DNA insertion line genotyping |
| AtbZIP18 Geno Fwd | GACAGCTTGAAAGTTTGGATAGACG | T-DNA insertion line genotyping |
| AtbZIP18 Geno Rev | CGAGCTTATCAGGAGCCATAGC | T-DNA insertion line genotyping |
| AtbZIP29 Geno Fwd | ATGGGTGATACAGAGAAGTGTAACAG | T-DNA insertion line genotyping |
| AtbZIP29 Geno Rev | CATTTGCCATGATCTTCTTCATTTCC | T-DNA insertion line genotyping |
| AtbZIP30 Geno Fwd | GATACAACAGATACCAATATGATGCAGAG | T-DNA insertion line genotyping |
| AtbZIP30 Geno Rev | CTTAGGGTCAGCCATTACAATCTC | T-DNA insertion line genotyping |
| AtbZIP33 Geno Fwd | TACGATACCTCCCATCTCTCG | T-DNA insertion line genotyping |
| AtbZIP33 Geno Rev | AGGCTGAGTCATAGACCGTG | T-DNA insertion line genotyping |
| AtbZIP52 Geno Fwd | TCGTTGAAGCAACTACAGAATCTTG | T-DNA insertion line genotyping |
| AtbZIP52 Geno Rev | TTCAGGAGGCATAGCTTTCTTAGC | T-DNA insertion line genotyping |
| PosF21 Geno Fwd | AATCTCATCGTCACAGTCACGG | T-DNA insertion line genotyping |
| PosF21 Geno Rev | CTCTTAGCACGTTTAGGATCAATGAG | T-DNA insertion line genotyping |
