# Supplementary material for: VIP1 and Its Homologs Are Not Required for Agrobacterium-Mediated Transformation, but Play a Role in Botrytis and Salt Stress Responses
Source: Front Plant Sci. 2018 Jun 12;9:749. doi: 10.3389/fpls.2018.00749 (PMC6005860; doi:10.3389/fpls.2018.00749)
Supplement: Supplementary file 3 [file Table_3.docx]

**Supplemental Table 3. *A. thaliana* lines used in this study**

| **Gene_ID** | **Name** | **Description** | **Source** |
| --- | --- | --- | --- |
| At1g43700 | *vip1-1* | Homozygous T-DNA insertion line | TAIR^1^: SALK_001014 |
| At1g43700 | *vip1-2* | Premature stop codon in first exon | This study |
| At1g43700/At2g31370/  At4g38900 | *vip1-1/posF21/bZIP29* | Triple mutant of three individual T-DNA insertion lines which were crossed to each other | Dr. Daisuke Tsugama (Tsugama et al., 2016) |
| At1g43700 | *VIP1-SRDX* Line #4, #7-1, and #11 | Transgenic lines overexpressing VIP1 fused to the SRDX repression domain | Dr. Daisuke Tsugama (Tsugama et al., 2016) |
| At1g43700 | Inducible *VIP1* Line #12 and #8 | Transgenic lines which inducibly overexpress VIP1 in the presence of β-estradiol | This study |
| At2g40620 | *bZIP18* | Homozygous T-DNA insertion line | TAIR: SALK_110712C |
| At4g38900 | *bZIP29* | Homozygous T-DNA insertion line | TAIR: SALK_065254C |
| At2g21230 | *bZIP30* | Homozygous T-DNA insertion line | TAIR: CS65514 |
| At2g12900 | *bZIP33* | Homozygous T-DNA insertion line | TAIR: SALK_054897C |
| At1g06850 | *bZIP52* | Homozygous T-DNA insertion line | TAIR: SALK_033320C |
| At2g31370 | *posF21* | Homozygous T-DNA insertion line | TAIR: SALK_024459C |
