# Supplementary material for: VIP1 and Its Homologs Are Not Required for Agrobacterium-Mediated Transformation, but Play a Role in Botrytis and Salt Stress Responses
Source: Front Plant Sci. 2018 Jun 12;9:749. doi: 10.3389/fpls.2018.00749 (PMC6005860; doi:10.3389/fpls.2018.00749)
Supplement: Supplementary file 4 [file Table_4.docx]

**Supplemental Table 4:** Real-time PCR results of two independent transgenic inducible *VIP1* lines.

|  | **Fold-change ± Standard Error** | | | |
| --- | --- | --- | --- | --- |
| **Inducible *VIP1***  **Line #12** | **Non-induced vs. Induced**  **3 hours** | **Non-induced vs. Induced**  **3 hours + *Agrobacterium*** | **Non-induced vs. Induced**  **12 hours** | **Non-induced vs. Induced**  **12 hours + *Agrobacterium*** |
| *VIP1* | 383.5 ± 134.7*** | 416.1 ± 65.7*** | 454.2 ± 62.1*** | 363.2 ± 92.9*** |
| *MYB44* | 1.8 ± 0.75 | 3.2 ± 0.8** | 2.5 ± 0.65*** | 2.0 ± 0.6*** |
| *PHI-1* | 2.9 ± 0.85*** | 3.7 ± 0.6 | 12.3 ± 1.75*** | 10.6 ± 2.85*** |
| *CYP707A1* | 2.0 ± 0.55*** | 1.9 ± 0.3 | 1.0 ± 0.15 | 1.9 ± 0.3*** |
| *CYP707A3* | 1.9 ± 0.5 | 2.4 ± 0.5*** | 4.2 ± 0.5** | 2.1 ± 0.6* |
| *MES1* | 2.0 ± 0.9 | 5.9 ± 1.75** | 3.0 ± 0.7* | 3.8 ± 1.65*** |
| *LYK3* | 0.6 ± 0.25 | 1.6 ± 0.95 | 6.4 ± 1.3* | 2.3 ± 0.8** |
| **Inducible *VIP1***  **Line #8** | **Non-induced vs. Induced**  **3 hours** | **Non-induced vs. Induced**  **3 hours + *Agrobacterium*** | **Non-induced vs. Induced**  **12 hours** | **Non-induced vs.**  **Induced**  **12 hours + *Agrobacterium*** |
| *VIP1* | 138.6 ± 7.85** | 94.5 ± 11.75*** | 267.4 ± 43.4 | 150.9 ± 21.65*** |
| *MYB44* | 1.5 ± 0.2* | 1.1 ± 0.1*** | 2.0 ± 0.45*** | 2.2 ± 0.2*** |
| *PHI-1* | 2.9 ± 0.25*** | 1.2 ± 0.1*** | 13.0 ± 2.0*** | 23.2 ± 2.85*** |
| *CYP707A1* | 1.2 ± 0.15 | 0.9 ± 0.2 | 1.3 ± 0.2 | 1.4 ± 0.15*** |
| *CYP707A3* | 1.5 ± 0.2*** | 1.0 ± 0.15 | 6.7 ± 1.3 | 3.0 ± 0.45*** |
| *MES1* | 2.1 ± 1.0 | 5.9 ± 1.75** | 3.9 ± 1.45* | 2.4 ± 1.25 |
| *LYK3* | 1.3 ± 0.85 | 1.6 ± 0.95 | 2.6 ± 0.55* | 3.5 ± 2.1* |

Changes in gene expression in induced versus non-induced samples are reported as the average fold-change of three technical replicates ± standard error. Significant P-values as determined by Student’s t-test are shown by astericks (*P-value < 0.05; **P-value < 0.01; ***P-value < 0.001).

**Supplemental references**

Citovsky V., Lee L-Y., Vyas S., Glick E., Chen M.H., Vainstein A., Gafni Y., Gelvin S.B., Tzfira T. (2006). Subcellular localization of interacting proteins by bimolecular fluorescence complementation *in planta*. *J Mol Biol* 362, 1120-1131.

Durfee T., Nelson R., Baldwin S., Plunkett G., Burland V., Mau B., Petrosino J.F., Qin X., Muzny

D.M., Ayele M., Gibbs R.A., Csörgo B., Pósfai G., Weinstock G.M., Blattner F.R. (2008). The

complete genome sequence of *Escherichia coli* DH10B: insights into the biology of the

laboratory workhorse. *J Bacteriol* 190, 2597-2606.

Hajdukiewicz P., Svab Z., Maliga P. (1994). The small, versatile pPZP family of *Agrobacterium*

binary vectors for plant transformation. *Plant Mol Biol* 25, 989-994.

Koncz C., Schell J. (1986). The promoter of TL-DNA gene 5 controls the tissue-specific expression

of chimeric genes carried by a novel type of *Agrobacterium* binary vector. *Mol Gen Genet* 204,

383-396.

Sciaky D.A., Montoya A.L., Chilton M-D. (1978). Fingerprints of *Agrobacterium* Ti plasmids.

*Plasmid* 1, 238-253.
