# Supplementary material for: VIP1 and Its Homologs Are Not Required for Agrobacterium-Mediated Transformation, but Play a Role in Botrytis and Salt Stress Responses
Source: Front Plant Sci. 2018 Jun 12;9:749. doi: 10.3389/fpls.2018.00749 (PMC6005860; doi:10.3389/fpls.2018.00749)
Supplement: Supplementary file 5 [file Image_1.pdf]

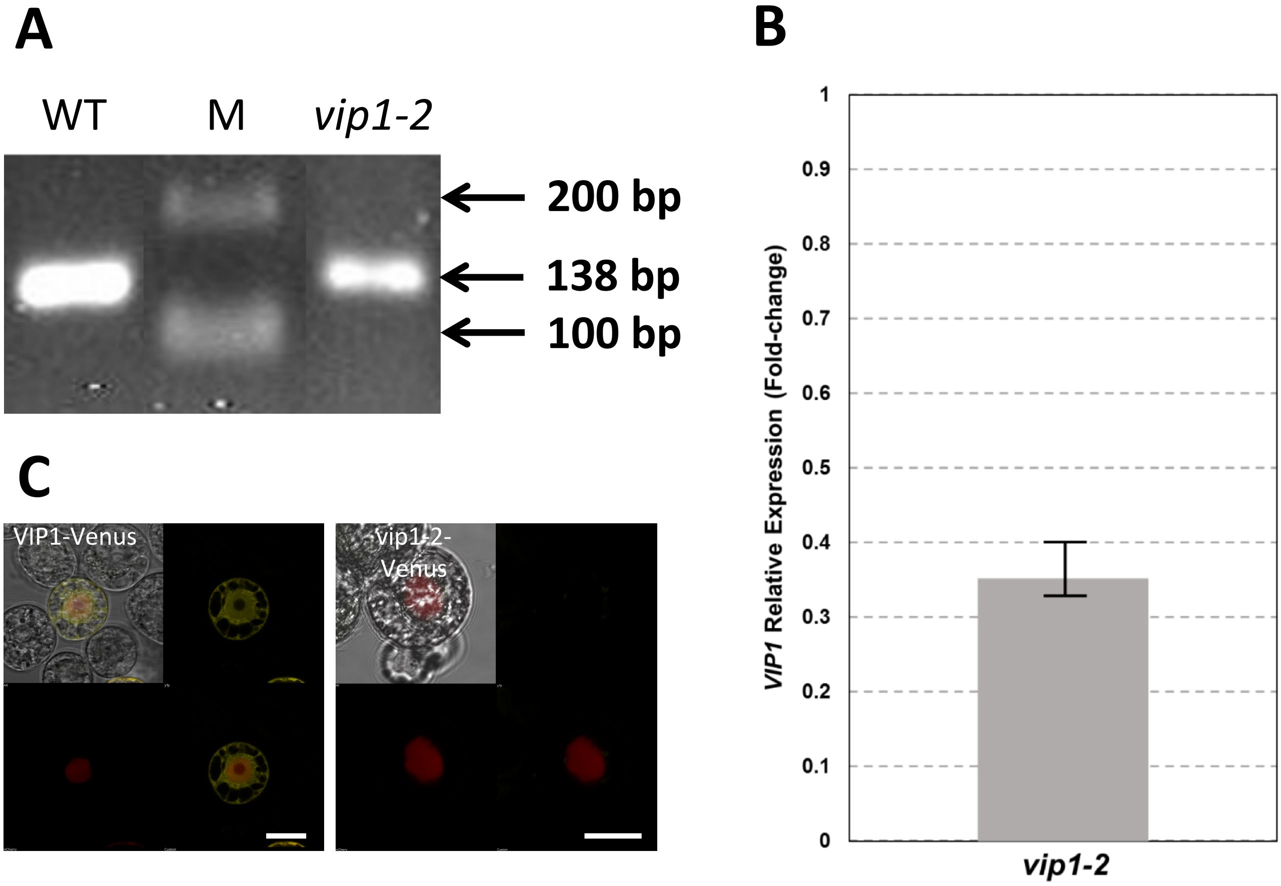

Supplemental Figure 1. RT-PCR and expression of wild-type (WT) Col-0 and *vip1-2* RNA. A. A 138 bp PCR product was amplified from the 3' end of the *VIP1 and vip1-2* transcripts and visualized by ethidium bromide staining after electrophoresis through a 1.5% agarose gel. M, size marker; **B.** Quantitative RT-PCR of *vip1-2* gene expression relative to that of VIP1 in wild-type plants. Results show the average of three technical replicates ± SE. **C.** Expression and localization of VIP-Venus (left) and vip1-2-Venus (right) in tobacco BY-2 protoplasts. Constructs comprised of cDNAs encoding VIP-Venus or vip1-2-Venus (vip1-2 out of frame with Venus) and a nuclear marker mRFP-NLS were co-transfected into tobacco BY-2. Transfected cells were imaged by confocal microscopy 16 hours later. Four images of each cell are presented (clockwise from top left: merged YFP, mRFP, and DIC; YFP; YFP + mRFP; mRFP). Bars indicate 20 mm.
