# Supplementary material for: VIP1 and Its Homologs Are Not Required for Agrobacterium-Mediated Transformation, but Play a Role in Botrytis and Salt Stress Responses
Source: Front Plant Sci. 2018 Jun 12;9:749. doi: 10.3389/fpls.2018.00749 (PMC6005860; doi:10.3389/fpls.2018.00749)
Supplement: Supplementary file 6 [file Image_2.pdf]

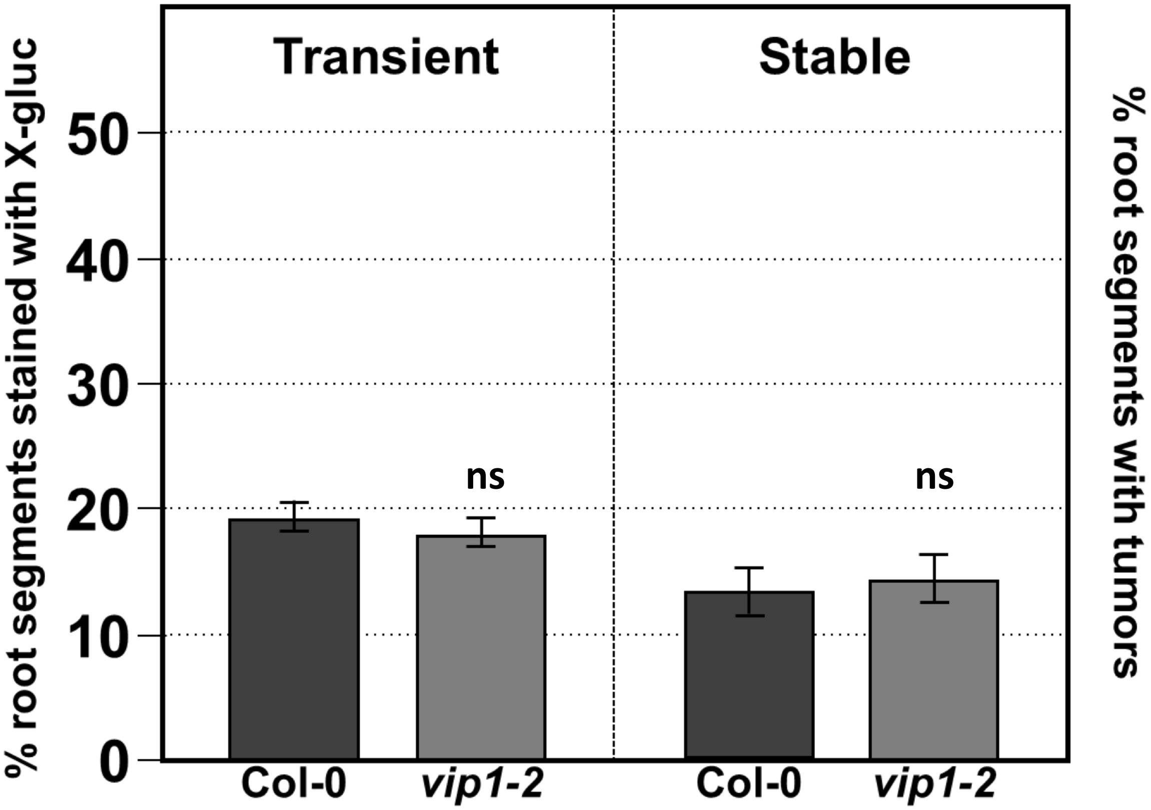

Supplemental Figure 2. Transformation susceptibility of *Arabidopsis* wild-type and *vip1-2* mutant plants.

Agrobacterium-mediated transient (left) or stable (right) transformation assays were conducted on wild-type and *vip1-2* mutant plants. Root segments were inoculated with 10^6 cfu/ml of the *A. tumefaciens strains* At849 (transient) or A208 (stable). For the transient assay, the root segments were stained with X-gluc 6 days after infection. For stable transformation, tumors were scored 30 days after infection. Numbers represent an average of three biological replicates (each replicate containing > 60 root segments) $\pm$ SE. Student's *t* test ns: not significant
