# Supplementary material for: VIP1 and Its Homologs Are Not Required for Agrobacterium-Mediated Transformation, but Play a Role in Botrytis and Salt Stress Responses
Source: Front Plant Sci. 2018 Jun 12;9:749. doi: 10.3389/fpls.2018.00749 (PMC6005860; doi:10.3389/fpls.2018.00749)
Supplement: Supplementary file 7 [file Image_3.pdf]

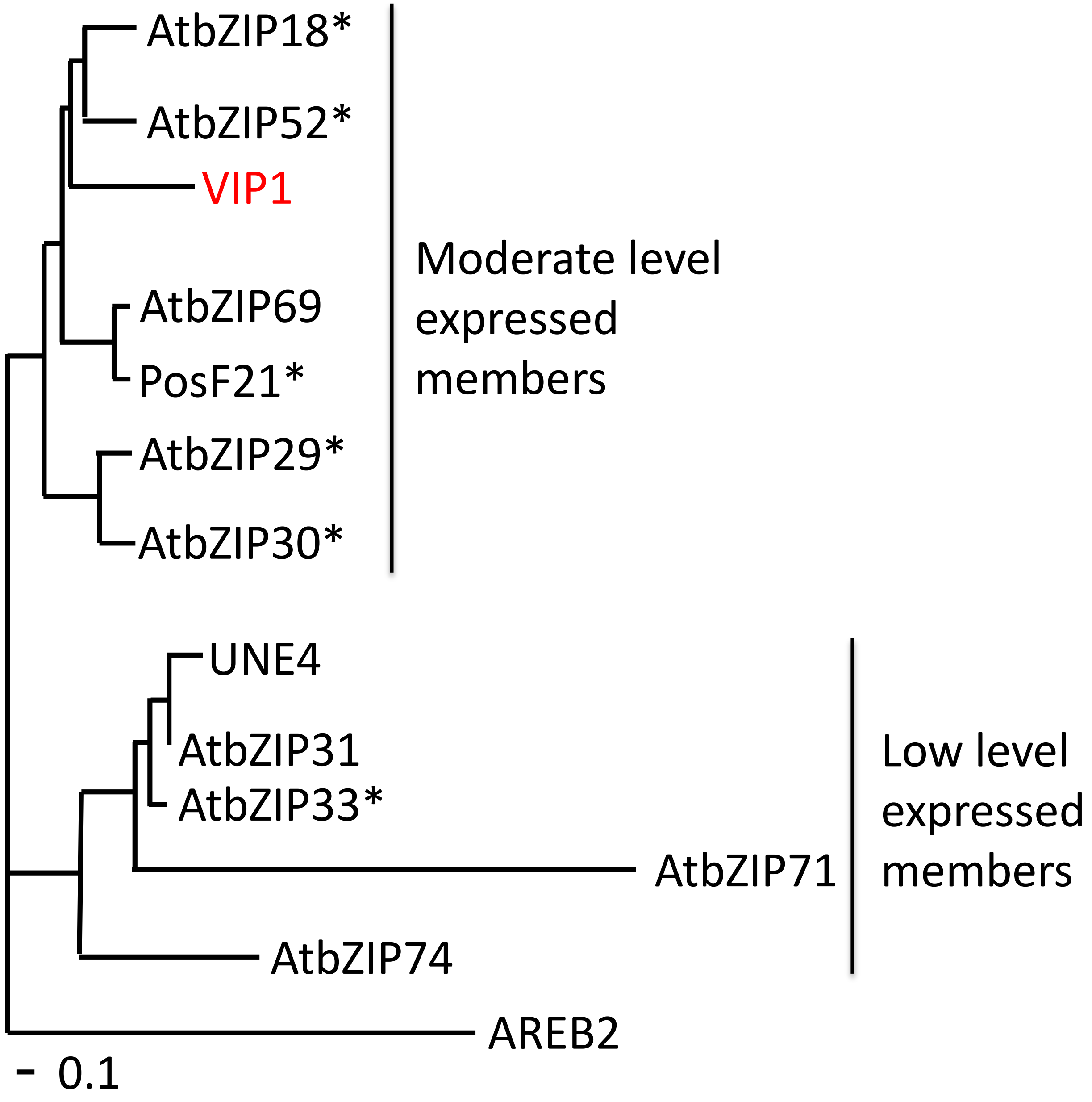

Supplemental Figure 3. Rooted phylogenetic tree of *Arabidopsis* group I bZIP proteins (Tsugama et al., 2014). Asterisks indicate null mutant lines of six VIP1 homologs that were tested for *Agrobacterium*-mediated transformation susceptibility. Bar: amino acid substitutions per site. Indicated expression levels are based on results of reverse transcription-PCR performed previously (Tsugama et al., 2014).
