# Supplementary material for: VIP1 and Its Homologs Are Not Required for Agrobacterium-Mediated Transformation, but Play a Role in Botrytis and Salt Stress Responses
Source: Front Plant Sci. 2018 Jun 12;9:749. doi: 10.3389/fpls.2018.00749 (PMC6005860; doi:10.3389/fpls.2018.00749)
Supplement: Supplementary file 9 [file Image_5.pdf]

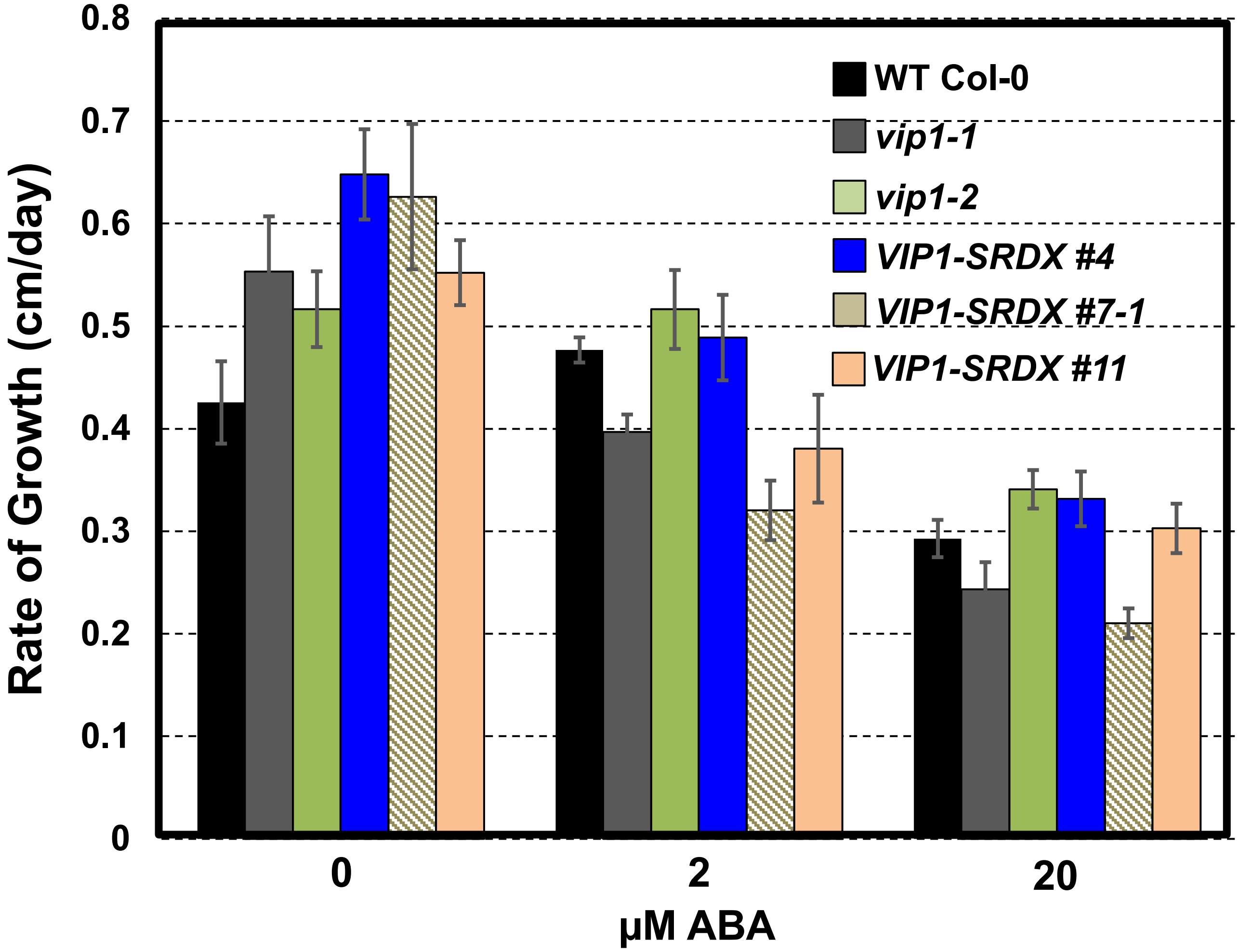

Supplemental Figure 5. Root growth rates of wild-type, *vip1* mutant, and VIP1-SRDX lines on various concentrations of ABA. Bars indicate the average rate of root growth ± SE. No mutant growth rate was significantly different from that of wild-type plants at any given ABA concentration.
