# Supplementary material for: VIP1 and Its Homologs Are Not Required for Agrobacterium-Mediated Transformation, but Play a Role in Botrytis and Salt Stress Responses
Source: Front Plant Sci. 2018 Jun 12;9:749. doi: 10.3389/fpls.2018.00749 (PMC6005860; doi:10.3389/fpls.2018.00749)
Supplement: Supplementary file 10 [file Image_6.pdf]

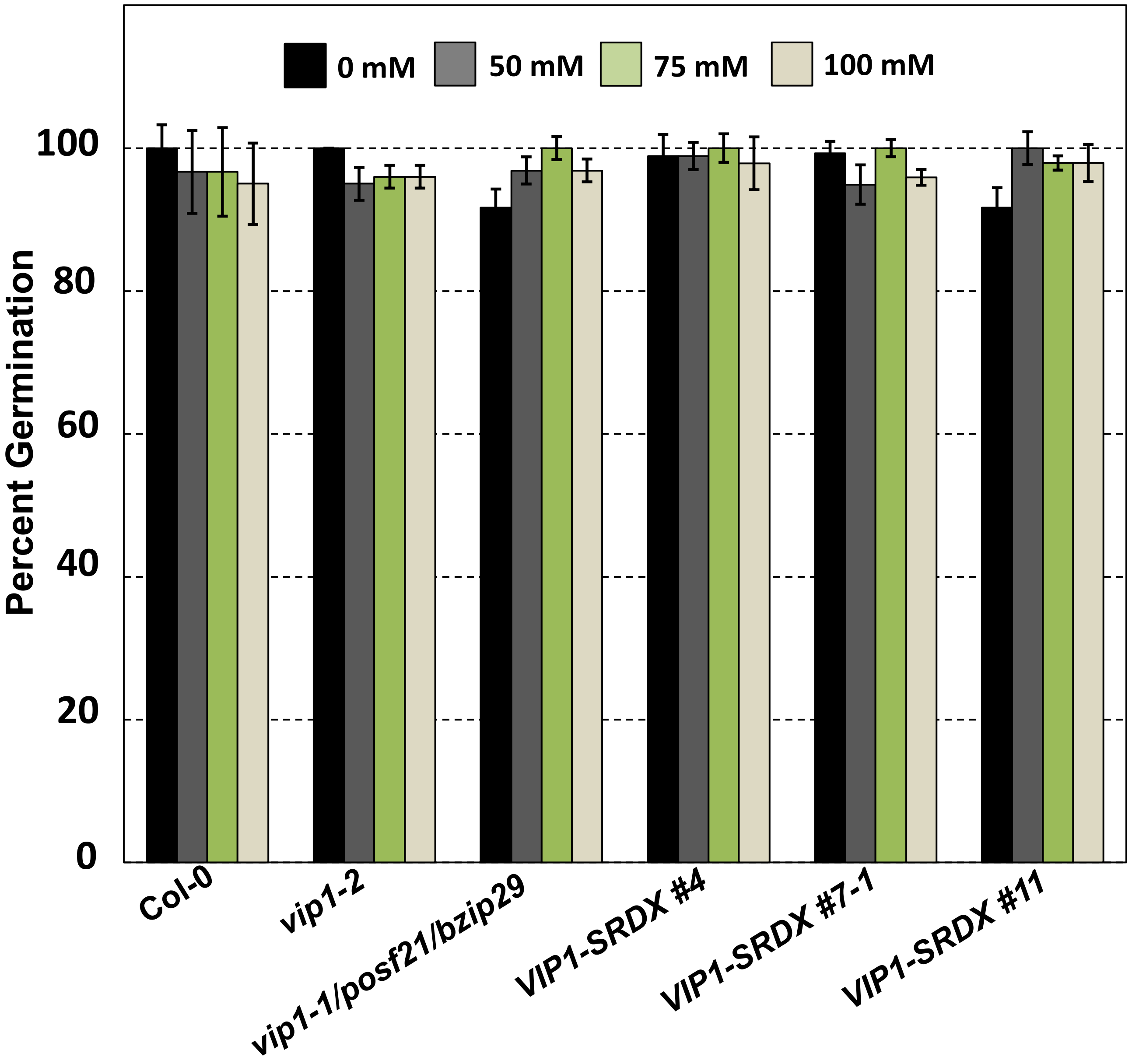

Supplemental Figure 6. Germination of wild-type, *vip1* mutants, and VIP1-SRDX lines on medium containing NaCl. Seeds of the indicated lines were germinated on B5 medium containing 0, 50, 75, or 100 mM NaCl. Bars indicate the average percent germination ± SE. No mutant germination rate was significantly different from that of wild-type plants at any given NaCl concentration.
